# Glucocorticoid Repression of Inflammatory Gene Expression Shows Differential Responsiveness by Transactivation- and Transrepression-Dependent Mechanisms

**DOI:** 10.1371/journal.pone.0053936

**Published:** 2013-01-14

**Authors:** Elizabeth M. King, Joanna E. Chivers, Christopher F. Rider, Anne Minnich, Mark A. Giembycz, Robert Newton

**Affiliations:** 1 Airways Inflammation Research Group, Snyder Institute for Chronic Diseases, Faculty of Medicine, University of Calgary, Calgary, Alberta, Canada; 2 Clinical Biomarkers Immunology, Bristol-Myers Squibb, Princeton, New Jersey, United States of America; National Heart and Lung institute, United Kingdom

## Abstract

Binding of glucocorticoid to the glucocorticoid receptor (GR/NR3C1) may repress inflammatory gene transcription via direct, protein synthesis-independent processes (transrepression), or by activating transcription (transactivation) of multiple anti-inflammatory/repressive factors. Using human pulmonary A549 cells, we showed that 34 out of 39 IL-1β-inducible mRNAs were repressed to varying degrees by the synthetic glucocorticoid, dexamethasone. Whilst these repressive effects were GR-dependent, they did not correlate with either the magnitude of IL-1β-inducibility or the NF-κB-dependence of the inflammatory genes. This suggests that induction by IL-1β and repression by dexamethasone are independent events. Roles for transactivation were investigated using the protein synthesis inhibitor, cycloheximide. However, cycloheximide reduced the IL-1β-dependent expression of 13 mRNAs, which, along with the 5 not showing repression by dexamethasone, were not analysed further. Of the remaining 21 inflammatory mRNAs, cycloheximide significantly attenuated the dexamethasone-dependent repression of 11 mRNAs that also showed a marked time-dependence to their repression. Such effects are consistent with repression occurring via the *de novo* synthesis of a new product, or products, which subsequently cause repression (i.e., repression via a transactivation mechanism). Conversely, 10 mRNAs showed completely cycloheximide-independent, and time-independent, repression by dexamethasone. This is consistent with direct GR transrepression. Importantly, the inflammatory mRNAs showing attenuated repression by dexamethasone in the presence of cycloheximide also showed a significantly greater extent of repression and a higher potency to dexamethasone compared to those mRNAs showing cycloheximide-independent repression. This suggests that the repression of inflammatory mRNAs by GR transactivation-dependent mechanisms accounts for the greatest levels of repression and the most potent repression by dexamethasone. In conclusion, our data indicate roles for both transrepression and transactivation in the glucocorticoid-dependent repression of inflammatory gene expression. However, transactivation appears to account for the more potent and efficacious mechanism of repression by glucocorticoids on these IL-1β-induced genes.

## Introduction

Glucocorticoids acting on the glucocorticoid receptor (GR/NR3C1) are the most effective anti-inflammatory drugs available for multiple inflammatory conditions [1]. Thus, inhaled glucocorticoids reduce lung inflammation and are consequently recommended for all but the mildest asthmatics [1], [2]. The profound anti-inflammatory effects of these drugs are largely derived from their ability to repress the expression of numerous inflammatory genes [3], [4]. While the classical paradigm of action for nuclear hormone receptors, such as GR, is to activate gene transcription from hormone response elements, e.g. palindromic glucocorticoid response elements (GREs), this has not generally been thought to explain repression of inflammatory gene expression [1], [4]. Rather this is attributed to multiple mechanisms of which transrepression, the ability of GR to directly repress gene transcription, is prominent [5]. In the transrepression hypothesis [6], ligand-activated GR interacts with, or tethers to, key transcription factors, such as NF-κB and AP–1, to switch off inflammatory gene transcription. GR may then recruit histone deacetylases (HDACs), in particular HDAC2 [7], to the promoters of inflammatory genes and thereby exert repression, in part, via effects on the local chromatin environment [8]. However, transrepression has also been explained by competition for co-activator molecules and GR-dependent changes in RNA polymerase II phosphorylation [9]–[11]. Equally, a dramatic glucocorticoid-dependent up-regulation of IκBα (NFKBIA), the endogenous inhibitor of NF-κB, was indicated as a major driver of glucocorticoid repression [12], [13]. However, many investigators have not observed such substantial effects and transcriptional repression of inflammatory genes can be dissociated from glucocorticoid-dependent increases in NFKBIA expression [14]–[17]. While other repressive mechanisms include GR binding to negative GREs (nGREs) in genes such as prolactin or proopiomelanocortin (POMC) [18], [19], a lack of readily definable nGRE sites in the promoters of inflammatory genes argued against this mechanism of repression [5]. Indeed, direct nGRE-dependent repression of POMC is probably described by tethering type interactions of GR at the promoter sites required for transcriptional activation [20]. Equally, GR chromatin immunoprecipitation (ChIP) experiments combined with genomic PCR, microarray (ChIP-chip) or high throughput sequencing (ChIP-SEQ) analyses primarily revealed GR binding sites at glucocorticoid-induced rather than repressed gene promoters [21]–[23]. Despite this, a more recent study has again raised the issue of nGREs as a widespread mechanism of repression [24]. While the degree to which these mechanisms apply in different systems remains to be fully investigated, it is essential to note that they cannot account for the now well established, but widespread, post-transcriptional and translational repression that is elicited by glucocorticoids on inflammatory gene expression [3], [6], [25].

In contrast to the above, we and many other investigators have noted that the ability of glucocorticoid to repress inflammatory gene expression is frequently prevented or attenuated by inhibition of transcription and/or translation (For examples see [3], [6], [25]). Rather than implying roles for “direct” mechanisms of transrepression, such data suggest that glucocorticoids induce the expression of “anti-inflammatory” genes to promote the repression of inflammatory gene expression [6], [26]. In this respect, glucocorticoids induce the expression of very many genes, for example NFKBIA, in multiple cell types [27]. While many such genes may play roles in the development of side-effects, it is clear that genes including mitogen-activated protein kinase (MAPK) phosphatase (MKP) 1 (DUSP1) or glucocorticoid-induced leucine zipper protein (GILZ) (TSC22D3) show biological properties that are consistent with repression of inflammatory gene expression [28], [29]. Thus DUSP1 is rapidly and robustly induced by glucocorticoids and may inactivate all three major MAPK pathways to reduce inflammatory responses via transcriptional, post-transcriptional, translational and post-translational effects [27], [28], [30]–[32]. Equally, TSC22D3 expression is increased in the lungs of asthmatics taking inhaled glucocorticoid, is variously described as a repressor of both AP-1 and NF-κB, and may also attenuate MAPK signalling [33]–[37].

The above indicates multiple mechanisms of glucocorticoid repression that may either be independent of a requirement for gene expression (i.e. classical transrepression) or dependent on gene expression (i.e. repression via the transactivation of anti-inflammatory genes). To explore contributions due to these (potentially competing) hypotheses, we examined the effect of the synthetic glucocorticoid, dexamethasone, on 39 inflammatory gene transcripts whose expression was induced by IL-1β, a representative inflammatory stimulus, in human pulmonary A549 cells. Since A549 cells and primary human bronchial epithelial cells both show enhanced inflammatory gene expression in the presence of IL-1β and repression by glucocorticoid [2], [38], [39], these cells represent a valid model to examine the relationship between repression by dexamethasone and the roles of transactivation and transrepression. In addition, the central role of NF-κB in transrepression models, led us to interrogate the role of this factor in the expression of IL-1β-induced genes.

## Materials and Methods

### Cell Culture and Drugs

A549 cells were grown to confluence in Dulbecco’s modified Eagle’s medium (DMEM) supplemented with 10% fetal calf serum and L-glutamine (all Invitrogen). Cells were incubated overnight in serum-free medium prior to the addition of fresh serum-free medium containing cytokine and drugs. IL-1β (R&D systems) was dissolved in phosphate-buffered saline plus 0.1% bovine serum albumin (both Sigma), dexamethasone (Sigma) was dissolved in Hanks’ balanced salt solution (HBSS), cycloheximide (Sigma) was dissolved in sterile water and ORG34517 (gift from Dr. Ard Peeters, Organon Laboratories, The Netherlands) was dissolved in DMSO. Final concentrations of DMSO were <0.1%.

### siRNA-mediated Gene Silencing

A549 cells at ∼60–70% confluence in 12 well plates were transfected with siRNAs. GR or control siRNA was mixed with Lipofectamine™ RNAi max (Invitrogen) (1 µg) in 100 µl of serum-free DMEM and then incubated at room temperature for 30 min. This mixture was then incubated on cells for 24 h at 37°C at a final siRNA concentration of 25 nM. Sequences for siRNA targeting were as follows: GR siRNA 6 (5′-AAGTGCAAACCTGCTGTGTTT-3′); Lamin siRNA (control) (5′-AACTGGACTTCCAGAAGAACA -3′) (both Qiagen).

### Adenoviral Infection, Luciferase Reporters and Assay

A549 cells at ∼70% confluence were incubated for 24 h in DMEM plus 10% fetal calf serum containing the indicated multiplicity of infection (MOI) of adenoviral serotype 5 (Ad5) vector, prior to incubating in serum-free medium overnight and then experimental treatments. Ad5-IκBαΔN encodes a dominant version of IκBα, while Ad5-NF-κB-luc contains five copies of the classical NF-κB motif [40]. Ad5-NF-κB-luc was introduced into A549 cells at a MOI of 1 and cells were harvested in 1× reporter lysis buffer (Biotium) 6 h after the addition of IL-1β. Luminescence was measured using a 20/20n Luminometer (Turner Biosystems).

### Western Blotting

Size-fractionation on 12% acrylamide gels, electroblotting to Hybond-ECL membranes (GE Healthcare) and immunodetection using ECL (Thermo Scientific) was as previously described [41]. Antibodies were: anti-IκBα/MAD-3 (M-18) (sc-1102, Santa Cruz), anti-GR (sc-8992, Santa Cruz) and anti-glyceraldehyde-3-phosphate (GAPDH) (4699–9555(ST), AbD Serotec).

### RNA Isolation, cDNA Synthesis and SYBR Green Real-time PCR

RNA was isolated and 0.5 µg reverse transcribed into cDNA [41]. After 1∶4 dilution, PCR was carried out on 2.5 µl of cDNA using SYBR GreenER mastermix (Invitrogen) and an ABI 7900HT instrument (Applied Biosystems). Relative cDNA concentrations were obtained from standard curves generated by serial dilution of an IL-1β-treated sample. Amplification conditions were: 50°C for 2 min, 95°C for 10 min then 40 cycles of 95°C for 15 s, 60°C for 1 min. For primer sequences see Table S1. Primer specificity was determined using dissociation (melt) curve analysis: 95°C for 15 s, 60°C for 20 s followed by ramping to 95°C over 20 min. A single peak in the change in fluorescence was taken to indicate specificity.

### Statistical Analysis

All numerical values are expressed to two significant figures. Graphical data are plotted as mean ± S.E. Statistical analysis between groups was performed using one-way analysis of variance (ANOVA) with a Bonferroni’s or Dunnet’s post test or a paired *t* test as indicated. Significance was assumed where: *, *p*<0.05; **, *p*<0.01; ***, *p*<0.001.

## Results

### Effect of IL-1β and Dexamethasone on Inflammatory Gene Expression

To identify genes whose expression was induced by IL-1β and modulated by dexamethasone, A549 cells were treated with maximally effective concentrations of IL-1β (1 ng/ml) and/or dexamethasone (1 µM). As we have described [42], [43], cells were harvested after 6 and 18 h and RNA subjected to microarray analysis using Affymetrix U95Av2 and B chips. This revealed 428 genes that were induced by >2-fold by IL-1β at 6 h (Table S2). While a small number of these IL-1β-induced genes, for example NFKBIA, PRIC285 and BIRC3, showed modest mRNA inducibility by dexamethasone alone, the majority revealed either no effect or repression of basal expression (Table S2, Figure S1). In the presence of IL-1β for either 6 or 18 h, heat maps of IL-1β-induced genes with ≥4 fold inducibility at 6 h showed the response to dexamethasone to vary in a gene-dependent manner with the overall effect of dexamethasone favouring repression (Figure S1A). As depicted in Figure S1C (Groups 1–5), three of these IL-1β-induced genes (SERPINA3, LOC645638, APOL1) revealed enhanced (≥1.25 fold) expression in the presence of dexamethasone (Group 1). In group 2, there were 38 genes showing either no, or modest (<1.25/≥0.75 fold), modulation of IL-1β-induced expression by dexamethasone, whereas groups 3, 4 and 5 contained genes with increasing repression by dexamethasone (Figure S1C). However, there was no significant relationship between the fold induction by IL-1β and the repression achieved by dexamethasone. (Figure S1D).

**Figure 1:**
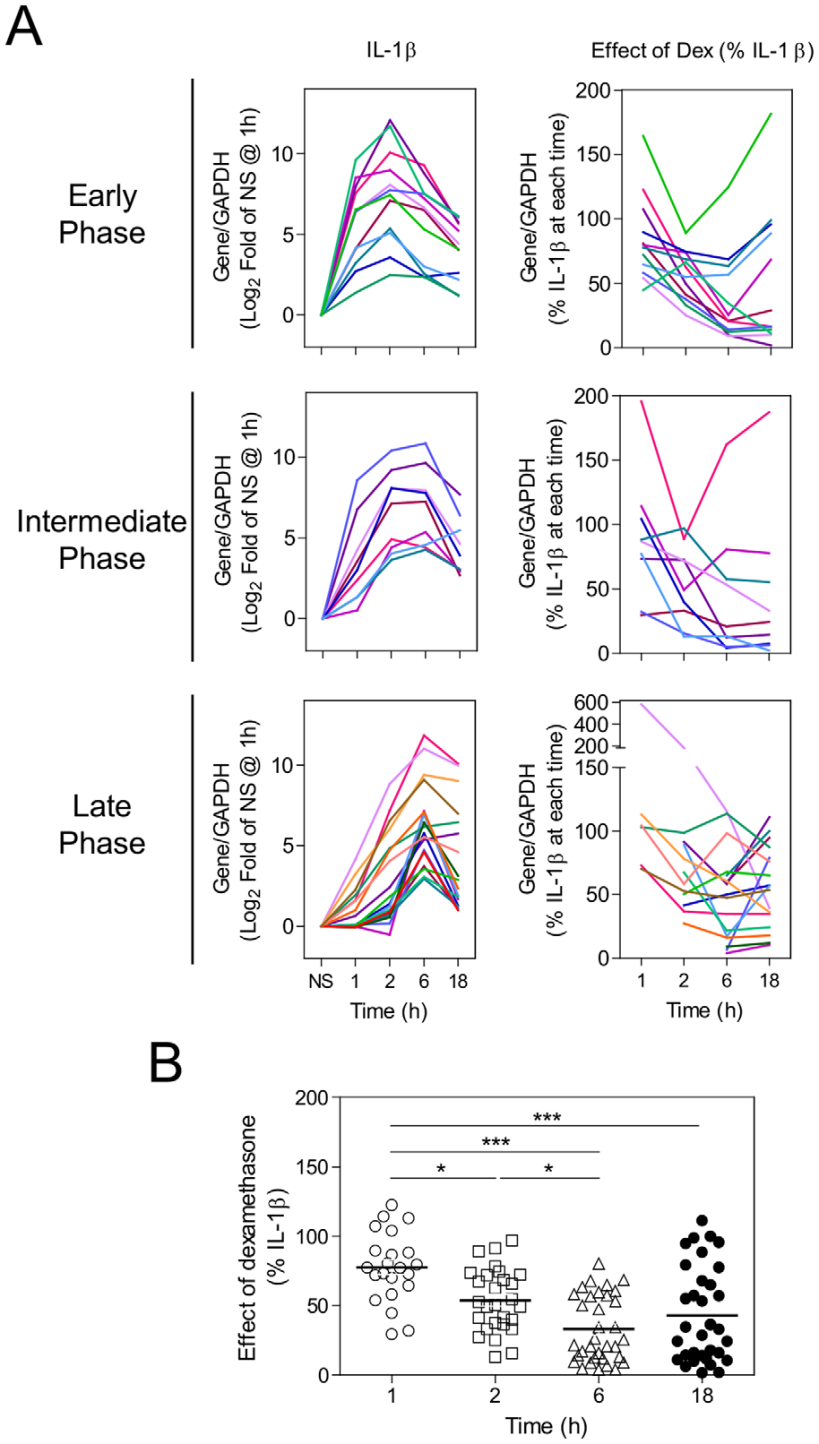
Effect of IL-1β and dexamethasone on inflammatory gene expression. A. A549 cells were either not stimulated (NS) or treated with IL-1β (1 ng/ml), dexamethasone (dex) (1 µM) or a combination of the two for 1, 2, 6 and 18 h. Cells were then harvested for RNA and real-time PCR was carried out for the indicated genes and GAPDH. Data (n = 3), normalised to GAPDH are expressed as either log_2_ fold over NS at 1 h (left panel) or as percentage of IL-1β (right hand panel) and plotted as means. Genes are grouped based on expression patterns: ‘Early-phase’ genes are those which have a peak of expression at 1 or 2 h (top two graphs); ‘Late-phase’ genes have a peak of expression at 6 h or later with less than 50% of that peak expression observed at 1 or 2 h (bottom two graphs); and ‘Intermediate’ genes are those that fall into neither of the above categories (middle two graphs). **B.** Effect of dexamethasone from right hand panel of A is plotted at each time point for all genes repressed by dexamethasone. Significance was tested using one-way ANOVA with a Bonferroni post-test and is indicated: *, *P*<0.05; ***, *P*<0.001.

To explore the regulation of IL-1β-induced genes by dexamethasone, 34 of the 35 genes corresponding to Affymetrix probe sets that individually showed >10-fold enhancement by IL-1β at 6 h were selected for analysis by real-time PCR (Table S2, Figure S1B). At the time of our analysis, the gene, c15orf48, was poorly annotated, did not show repression by dexamethasone (Figure S1A) and was not analysed. In addition, four commonly studied inflammatory genes, GM-CSF (CSF2), COX-2 (PTGS2), IL-1β (IL1B) and TNFα (TNF), were also selected for analysis. In each case, a single primer pair crossing an exon/exon boundary was designed for each gene to amplify all RefSeq splice variants, with the exception of IFIT3, which has two splice variants with only one exon in common. Therefore, two primer pairs were designed for this gene and designated IFIT3iso1 and IFIT3iso2. A total of 39 mRNAs were subjected to real-time PCR analysis. A549 cells were treated with IL-1β and dexamethasone, either alone or in combination, for 1, 2, 6 or 18 h. The mRNA expression profiles for these 39 mRNAs were placed into three categories (early, intermediate and late - see figure legends for definitions) based on the timing of peak expression induced by IL-1β as represented by the line graphs in Figure 1A (for detailed information on each individual gene see Figure S2).

Of the 39 IL-1β-induced transcripts analysed by PCR, twelve, including PRIC285 and BIRC3, which were modestly dexamethasone induced in the array (Figure S1), showed two or more fold enhancement by dexamethasone alone for at least one time point (Table 1, Figure S2) and this effect was significant for seven genes. For example, dexamethasone induced CSF3 mRNA by up to 30 fold at all time points. Although lower in magnitude, similar responses were seen for BIRC3, TNFAIP3 and G0S2, thus identifying these genes as *bona fides* dexamethasone-induced genes. Conversely, and despite significant increases in mRNA for at least one time point, the effects on IFIT1, LAMB3 and PRIC285 were less consistent. While the functional relevance of these observations requires consideration, the induction by dexamethasone was considerably less than the maximal induction by IL-1β (Table 1).

**Table 1:** Effect of dexamethasone treatment alone on inflammatory mRNA expression.

		1 h		2 h		6 h		18 h	
Gene	Max Inductionby IL-1β (Fold)	Induction byDex (Fold)	% IL-1β	Induction byDex (Fold)	% IL-1β	Induction byDex (Fold)	% IL-1β	Induction byDex (Fold)	% IL-1β
**BIRC3**	30	2.0	**6.6**	4.7*	**16**	4.9*	**16**	5.7	**19.0**
**CFB**	54	1.1	**2.0**	0.93	**1.7**	1.4	**2.6**	3.8	**7.0**
**CSF3**	2100	15*	**0.75**	31***	**1.5**	20**	**1.0**	19	**0.89**
**CXCL2**	510	3.7	**0.72**	1.1	**0.22**	1.8	**0.36**	2.8	**0.56**
**EFNA1**	12	0.62	**5.3**	0.39	**3.3**	0.81	**6.8**	2.0	**17**
**G0S2**	41	1.02	**2.5**	1.8	**4.3**	5.2*	**13**	5.4	**13**
**CSF2**	4300	1.5	**0.035**	2.9	**0.067**	1.5	**0.034**	0.19	**0.0045**
**IFIT1**	26	1.2	**4.6**	1.5	**5.7**	2.1**	**7.8**	3.5*	**13**
**IL32**	87	1.2	**1.3**	1.3	**1.5**	2.1	**2.4**	1.8	**2.1**
**LAMB3**	12	0.88	**7.4**	1.09	**9.2**	1.5	**13**	2.6*	**22**
**PRIC285**	24	0.94	**3.9**	1.5	**6.0**	2.8*	**11**	2.2	**8.9**
**TNFAIP3**	170	3.2*	**1.8**	3.5*	**2.0**	3.4*	**2.0**	3.1	**1.8**

A549 cells were either not treated or stimulated with IL-1β (1 ng/ml), dexamethasone (Dex) (1 µM) or a combination of the two for 1, 2, 6 or 18 h prior to harvesting for real-time PCR analysis of 39 inflammatory genes and GAPDH. Data (n = 3) normalised to GAPDH are presented as means ± SE for genes showing greater than 2-fold induction by dexamethasone at any time point. Significance relative to non-stimulated samples was tested at time points showing greater than 2-fold induction using a paired t-test and is indicated:

* , *P*<0.05;

** , *P*<0.01;

*** , *P*<0.001.

**Figure 2:**
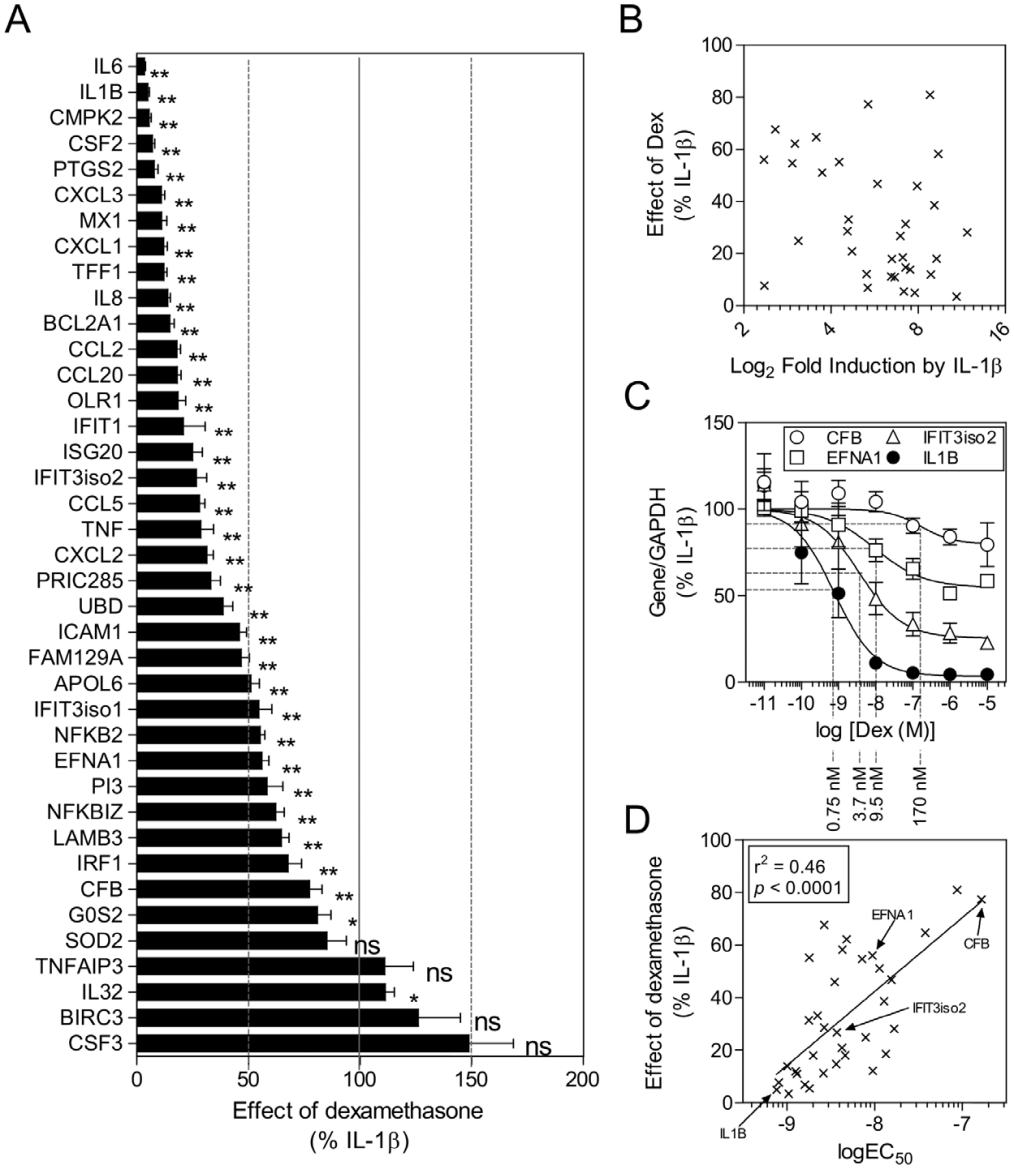
Effect of dexamethasone on inflammatory gene expression. **A.** The effect of dexamethasone (1 µM) is shown on the induction of inflammatory genes by IL-1β (1 ng/ml) at 6 h. Data from Figures S2 and S3 were combined and the effect of dexamethasone expressed as a percentage of IL-1β for each gene. 100% indicates no effect of dexamethasone. Data (n = 9) are plotted as means ± SE. Genes are listed by descending efficacy to repression by dexamethasone. Statistical analysis was performed by non-parametric paired t-test. * *P*<0.05; ** *P*<0.01. **B.** Relationship between induction by IL-1β and repression by dexamethasone. The effect of dexamethasone (1 µM) expressed as a percentage of IL-1β is plotted against the fold induction of each gene. Data are derived from Figure 2A and Figure S2. Linear regression was performed using GraphPad Prizm software. **C.** Repression by dexamethasone is shown for the most highly sensitive gene (IL-1β), two genes showing intermediate sensitivity (EFNA1 & IFIT3 isoform 2) and the lowest sensitivity gene (CFB) (that showed significant repression by 1 µM dexamethasone). Actual EC_50_ values are indicated. **D.** Relationship between the effect of 1 µM dexamethasone (efficacy) and the sensitivity (log EC_50_) of the repression by dexamethasone for individual genes. The effect of dexamethasone (1 µM) expressed as percentage of IL-1β is plotted against the log EC_50_ for the repression of each gene. Data are derived from Figure 2A and Figure S3 respectively. Linear regression was performed using GraphPad Prizm software.

In combination with IL-1β, dexamethasone showed varying effects on inflammatory gene expression (Figure 1, Figure S2). Analysis of the effect of 1 µM dexamethasone on IL-1β-induced mRNA at 6 h showed that 34 of the IL-1β-inducible mRNAs were significantly repressed by dexamethasone, whereas five genes, (BIRC3, CSF3, IL32, SOD2 and TNFAIP3) were not repressed (Figure 2). Furthermore, of the mRNAs repressed by dexamethasone, the majority showed increasing levels of repression with time, a trend which is readily seen in the line graphs in Figure 1A (for detailed information on individual genes see Figure S2). For example, at 1 h, repression of CCL2, CCL20, PTGS2 and others was minimal, yet increased markedly by 2 and 6 h (Figure S2). Indeed, after excluding the 5 genes not repressed by dexamethasone, the combined repression of all genes by dexamethasone was significantly greater at 2 h and 6 h when compared to 1 h (Figure 1B). Equally, the overall level of repression was significantly greater at 6 h compared to 2 h; thus, a clear time-dependence to the repression of most inflammatory genes by dexamethasone is established.

### Effect of Dexamethasone Concentration on IL-1β-induced Gene Expression

A549 cells were treated with IL-1β and various concentrations of dexamethasone for 6 h prior to real-time PCR analysis of all 39 mRNAs (Figure S3). While IL-1β-induced expression of BIRC3, CSF3 and IL32 appeared to be elevated in the presence of increasing dexamethasone concentrations, this was only significant in the case of IL32 and there was no obvious effect on SOD2 or TNFAIP3 (Figure 2A). As with the array data, repression by dexamethasone did not correlate with induction by IL-1β (Figure 2B). Analysis of the EC_50_ values for the 34 genes repressed by dexamethasone suggested that mRNAs showing the greatest level of repression (i.e. greatest E_max_) may also show the highest potency (i.e. lowest EC_50_) to dexamethasone (Figure S3, Figure 2C). Thus, CFB was the least repressed mRNA and with an EC_50_ of 170 nM was also the least potently repressed gene. Conversely, IL1B, the mRNA most strongly repressed by dexamethasone, was also the most potently repressed mRNA with EC_50_ of 0.75 nM. Transcripts such as EFNA1 and IFIT3iso2 showed lower levels of repression and dexamethasone had intermediate potency. Furthermore, a highly significant (*P*<0.0001) correlation between maximal efficacy and log potency of dexamethasone-mediated repression amongst genes was observed, thus confirming this observation (Figure 2D, Table S3).

### Repression of IL-1β-induced Gene Expression by Dexamethasone is Dependent on GR

**Figure 3:**
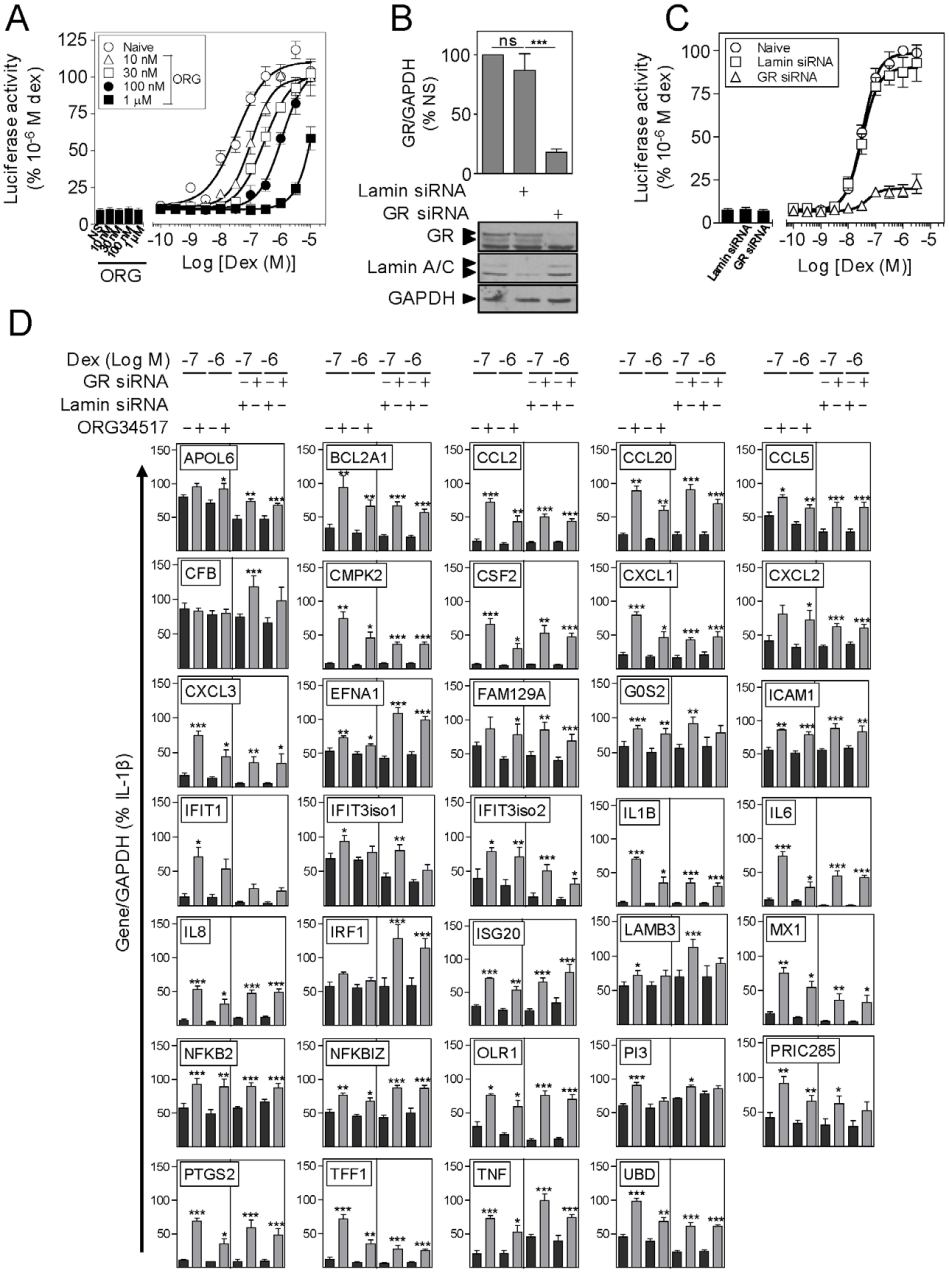
Effect of ORG34517 and GR-specific siRNA on repression of inflammatory gene expression by dexamethasone. A. A549 cells stably transfected with a 2×GRE reporter were incubated with the indicated concentrations of ORG34517 for 30 min prior to stimulation with increasing concentrations of dexamethasone (Dex) as indicated for 6 h. Cells were then harvested for luciferase assay. Data (n = 4–5) are expressed as a percentage of 1 µM dexamethasone and plotted as means ± SE. **B.** A549 cells were incubated with either lamin- (control) or GR-specific siRNA for 24 h prior to harvesting for western blot analysis of lamin A/C, GR and GAPDH. Following densitometric analysis, data (n = 5), normalised to GAPDH and expressed as percentage of NS are plotted as means ± SE. Significance, relative to lamin siRNA treated cells, using ANOVA with a Dunnet’s post-test, is indicated: ns, not significant; ***, *P*<0.001. Representative blots are shown. **C.** A549 cells stably transfected with a 2×GRE reporter were incubated with lamin- (control) or GR-specific siRNA for 24 h prior to stimulation with increasing concentrations of dexamethasone (Dex) as indicated. After 6 h, cells were harvested for luciferase assay. Data (n = 4–5) are expressed as percentage of 1 µM dexamethasone and plotted as means ± SE. **D.** A549 cells were either not treated or incubated with either ORG34517 for 30 min (left panel of each graph), or lamin- (control) or GR-specific siRNA for 24 h (right panel of each graph) prior to being either not stimulated (data not shown), or treated with IL-1β (1 ng/ml) in the absence (data not shown) or presence of either 0.1 or 1 µM dexamethasone (Dex) for 6 h. Cells were then harvested for real-time PCR analysis of the indicated genes and GAPDH. Data (n = 5–6) normalised to GAPDH and expressed as percentage of IL-1β are plotted as means ± SE. Left panel of each graph: significance relative to IL-1β+Dex was tested using a paired t-test and is indicated: *, *P*<0.05; **, *P*<0.01; ***, *P*<0.001. Right panel of each graph: significance relative to IL-1β+Dex+Lamin siRNA was tested using ANOVA with a Dunnett’s post test (see also Table S4) and is indicated: *, *P*<0.05; **, *P*<0.01; ***, *P*<0.001.

To evaluate the extent to which repression of IL-1β-induced gene expression by dexamethasone was dependent on GR, two complementary approaches were undertaken. Firstly, the effects of a competitive GR antagonist, ORG34517, which compared to RU486 shows reduced partial agonism [44] (Figure 3A), and secondly siRNA targeting of GR (Figures 3B and 3C), were tested on A549 cells harbouring a simple 2×GRE-luciferase reporter prior to analysis of IL-1β-induced mRNAs in the presence of dexamethasone (Figure 3D).

In response to dexamethasone, 2×GRE-dependent luciferase activity was concentration-dependently increased (EC_50_ = 34 nM) (Figure 3A). Treatment with increasing concentrations of ORG34517 prior to addition of dexamethasone produced a rightward, parallel displacement of the concentration-curve that described GRE-dependent transcription, without significantly affecting the maximum response (Figure 3A). These data are indicative of competitive antagonism and a pA_2_ of 8.5 and Schild slope of 1.07 was obtained using the Gaddum-Schild equation, as previously described [45]. Since luciferase activity induced by 1 µM dexamethasone was essentially prevented by 1 µM ORG34517, A549 cells were treated with 1 µM ORG34517 for 30 min prior to stimulation with IL-1β and dexamethasone (1 and 0.1 µM). Real-time PCR analysis revealed that the dexamethasone-dependent repression of 32 out of the 34 inflammatory mRNAs was significantly reversed by ORG34517 (Figure 3D), supporting the role of GR. Of the two genes not showing reversal, CFB was so modestly repressed by dexamethasone that effects of ORG34517 would be difficult to identify. Equally, IRF1, which is partially repressed by dexamethasone, showed reversal by ORG34517, but this failed to reach significance. In addition, while ORG34517 (alone) had no effect on IL-1β-induced mRNA expression of 31 genes, significant, but minor, repression of CCL2, PTGS2 and TFF1 was observed and is indicative of partial agonism (Table S4).

Transfection of a lamin-specific control siRNA knocked-down expression of lamin A/C, but had no significant effect on GR protein expression (Figure 3B). Conversely, a GR-specific siRNA inhibited expression of GR by 82±2.9%, without affecting lamin A/C expression (Figure 3B). Parallel experiments examining dexamethasone-dependent transcription in 2×GRE reporter cells revealed no effect of the lamin siRNA, whereas the GR-specific siRNA strongly attenuated reporter activity (Figure 3C). A549 cells were therefore transfected with lamin or GR-specific siRNA prior to stimulation with IL-1β and 0.1 or 1 µM dexamethasone. Real-time PCR analysis showed a significant reversal of repression, for 33 out of the 34 genes, by 0.1 and/or 1 µM dexamethasone in the presence of the GR-specific, but not lamin-specific siRNA (Figure 3D). In respect of PRIC285, a marked loss of dexamethasone-dependent repression was observed in the presence of GR-specific siRNA, but this did not reach statistical significance. Since, with the exception of G0S2, there was no effect of lamin siRNA on the IL-1β plus dexamethasone-induced expression of any of these mRNAs (Table S4), these data support a role for GR only in the repression elicited by dexamethasone.

### NF-κB Dependence of IL-1β-induced Genes

**Figure 4:**
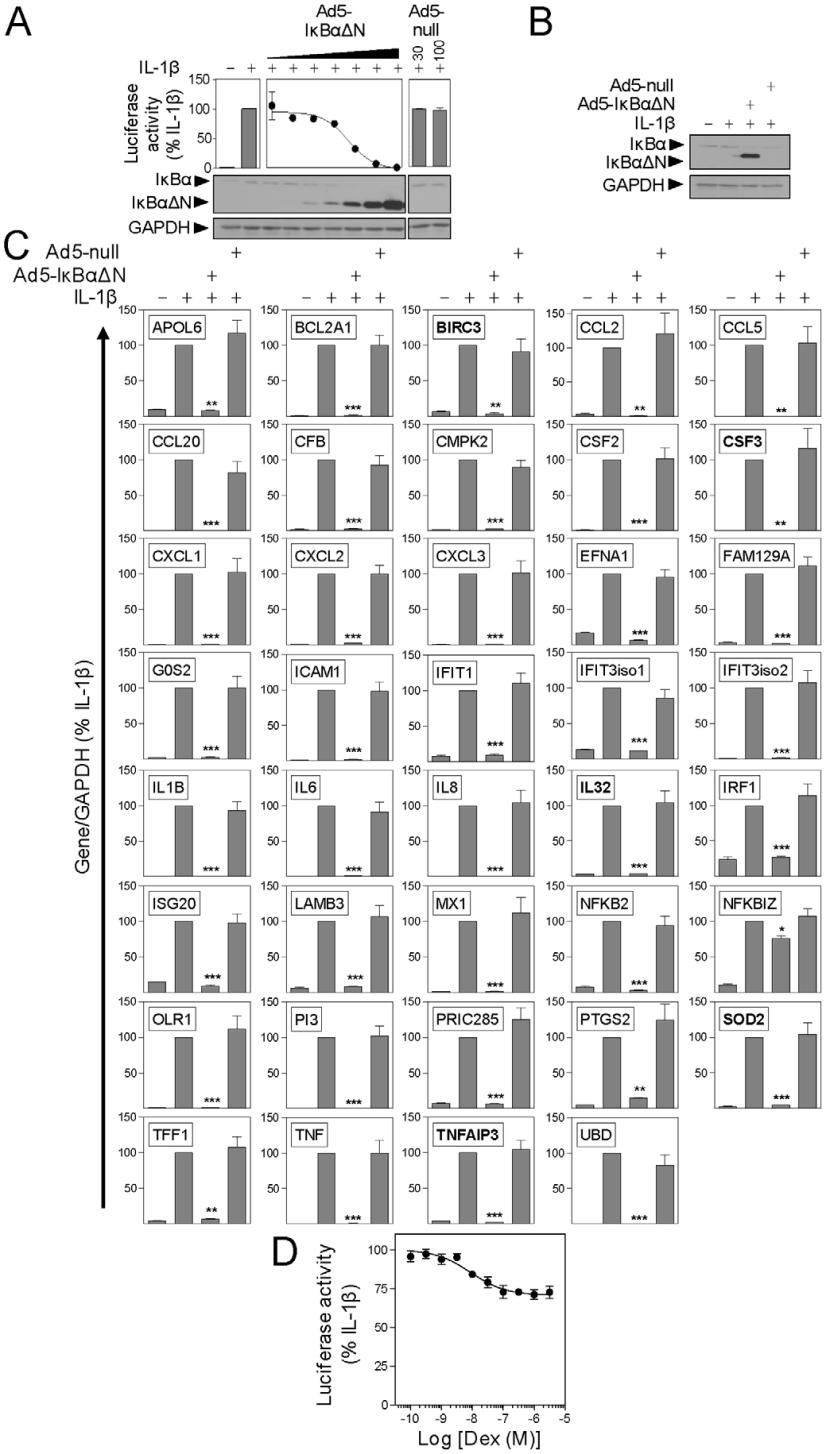
Effect of IκBαΔN on inflammatory gene expression. **A.** A549 cells were infected with the NF-κB-dependent reporter, Ad5-NF-κB-luc, in the presence of Ad5-IκBαΔN (0.1–100 MOI) or Ad5-null. After 36 h, cells were stimulated with IL-1β (1 ng/ml) for 6 h before harvesting for luciferase assay and western blot analysis of IκBα and GAPDH. Data (n = 2) expressed as percentage of IL-1β are plotted as mean ± SE. Representative blots are shown. **B.** A549 cells were infected with 100 MOI of Ad5-IκBαΔN or Ad5-null for 24 h prior to stimulation with IL-1β for 6 h. Cells were harvested for western blot analysis of IκBα and GAPDH. Blots representative of 4 experiments are shown. **C.** Cells from B were harvested for RNA and SYBR green real-time PCR carried out for GAPDH and the indicated genes. Data (n = 4) normalised to GAPDH and expressed as percentage of IL-1β are plotted as mean ± S.E. Significance relative to IL-1β was tested using AVOVA with a Dunnett’s post-test. * *P*<0.05; ** *P*<0.01; *** *P*<0.001. Genes not significantly repressed by dexamethasone (from Figure 2A) are highlighted in bold font. **D.** A549 cells infected with the NF-κB-dependent reporter, Ad5-NF-κB-luc, for 36 h were incubated with various concentrations of dexamethasone, as indicated, for 1 h prior to stimulation with IL-1β. After 6 h cells were harvested for luciferase assay. Data (n = 5) expressed as percentage of IL-1β are plotted as mean ± SE.

In the presence of increasing MOIs of the dominant NF-κB inhibitor, Ad5-IκBαΔN [40], overexpression of IκBαΔN was observed (Figure 4A). This correlated with repression of NF-κB-dependent transcription and the inhibition of endogenous IκBα protein expression as previously reported (Figure 4A) [46]. Since maximal effects were achieved at MOI 100, with no effect of a control virus, this concentration was selected for analysis of IL-1β-induced gene expression. As before, overexpression of IκBαΔN was confirmed and this correlated with reduced endogenous expression of IκBα (Figure 4B). Inflammatory gene expression analysed in parallel experiments revealed IL-1β-induced expression of all 39 mRNAs to be significantly prevented by Ad5-IκBαΔN (Figure 4C). In all cases, with the exception of NFKBIZ, where inhibition was modest, IL-1β-induced mRNA expression was reduced to near basal levels by Ad5-IκBαΔN (Figure 4C). Therefore, the effect of NF-κB inhibition does not correlate with repression by dexamethasone. (Figure S4). This was most obvious in respect of the 5 genes that were not repressed by dexamethasone (BIRC3, CSF3, IL32, SOD2 and TNFAIP3), but which were highly NF-κB-dependent (Figure 4C). Additionally, analysis of an NF-κB-dependent luciferase reporter, Ad5-NF-κB-luc, revealed only a partial, 29% repression by dexamethasone (Figure 4D).

### Effect of Cycloheximide on Dexamethasone-dependent Repression

Classical transrepression occurs via a direct mechanism that is insensitive to protein synthesis inhibition [5], whereas repression that depends on the transactivation of glucocorticoid-inducible genes such DUSP1, TSC22D3 or others will be prevented by protein synthesis inhibition. Therefore, the effect of cycloheximide, a protein synthesis inhibitor, was explored on the dexamethasone-dependent repression of the inflammatory mRNAs.

A549 cells were treated with IL-1β in the absence or presence of cycloheximide and/or dexamethasone. After 4 h, cells were harvested for real-time PCR analysis of inflammatory gene expression. In the presence of cycloheximide, the IL-1β-induced expression of 13 of the inflammatory gene mRNAs was significantly inhibited (Figure S5). We interpret these data as showing that these are second phase genes, i.e. their expression is dependent on the prior expression of other factors, for example inducible transcription factors, and this assessment is supported by the prior demonstration of ‘late-phase’ kinetics (Figure 1, Figure S2). As a consequence, these genes, along with the 5 genes not repressed by dexamethasone (Figure 2A), were excluded from further analysis. Thus our current analysis is necessarily limited to evaluating the role of ongoing protein synthesis in the repression of the early phase inflammatory genes by dexamethasone.

**Figure 5:**
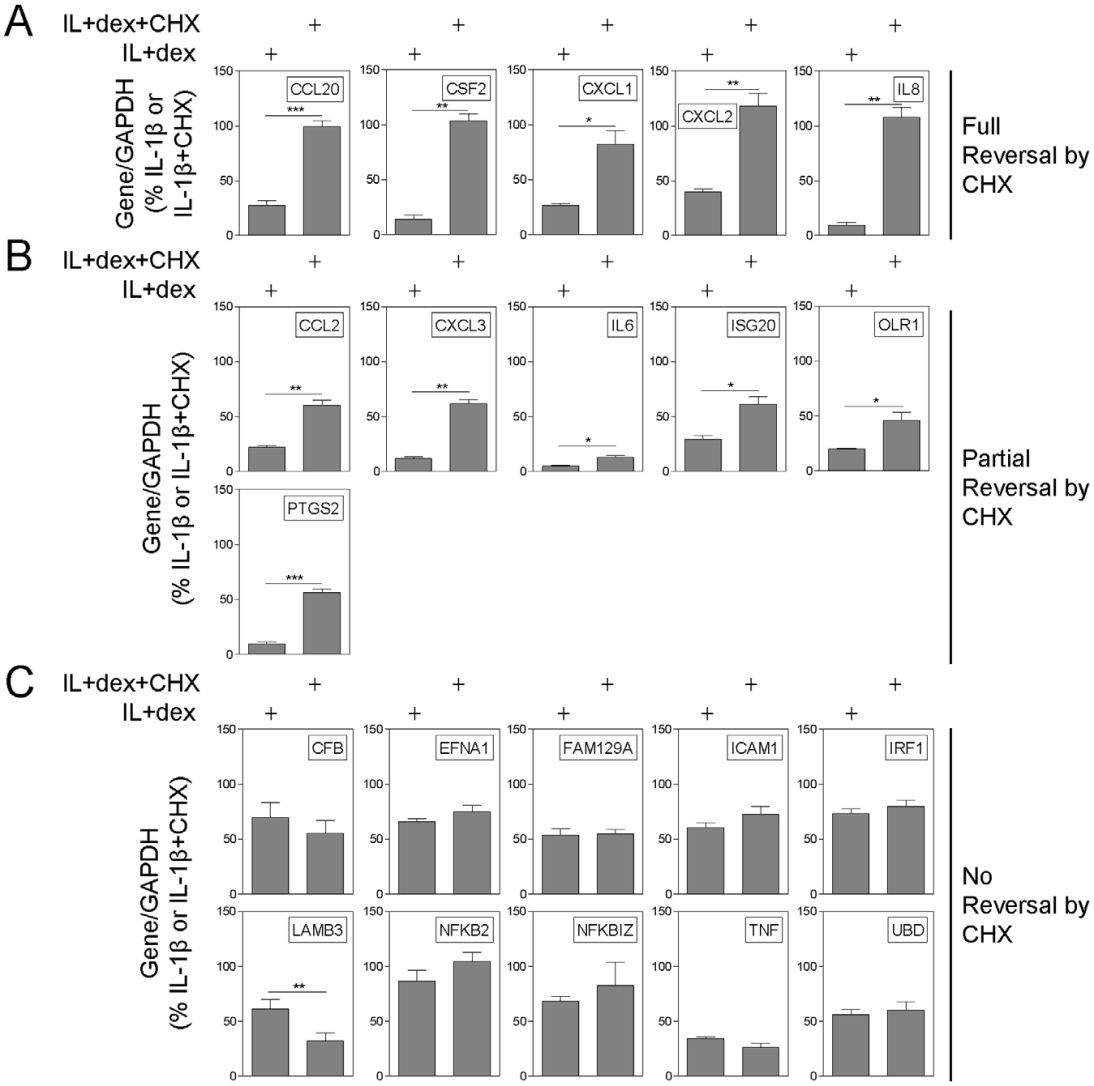
Effect of CHX on dexamethasone-dependent repression of inflammatory gene expression. A549 cells were treated with IL-1β (1 ng/ml) (not shown), or IL-1β and dexamethasone (dex) (1 µM) in the absence or presence of cycloheximide (CHX) (100 µg/ml) as indicated, for 4 h. Cells were then harvested for RNA and real-time PCR carried out for the indicated genes and GAPDH. Data (n = 4) normalised to GAPDH and plotted as percentage of IL-1β or IL-1β+CHX are expressed as mean ± S.E. Significance, relative to IL-1β+dex, using a paired t-test is indicated; *, *P*<0.05; **, *P*<0.01; ***, *P*<0.001. **A.** Full reversal (significantly different from IL-1β+dex and from IL-1β+CHX), **B.** partial reversal (significantly different from only IL-1β+dex) and **C.** no reversal (not significantly different from IL-1β+dex) of dexamethasone-dependent repression by CHX.

For the remaining 21 IL-1β-induced mRNAs, cycloheximide had one of three effects on the repression elicited by dexamethasone. Cycloheximide significantly blocked the dexamethasone-dependent repression of 11 genes (Figure 5). These can be classified into two groups: i) mRNAs for which cycloheximide “fully reversed” the repression by dexamethasone (Figure 5A), and; ii) mRNAs where there was only a “partial reversal” of the dexamethasone-dependent repression by cycloheximide (Figure 5B). “Full reversal” was defined as where the percentage repression by dexamethasone was significantly reversed in the presence of cycloheximide and resulted in no significant repression, i.e. there was no difference from control (IL-1β plus cycloheximide). Similarly, “partial reversal” is when the percent repression by dexamethasone was significantly reversed by cycloheximide, but that this was still significantly repressed compared to control (IL-1β plus cycloheximide). i.e. repression still occurred, but to a lesser extent. Finally, there was a group of 10 mRNAs showing “no reversal” for which the presence of cycloheximide did not attenuate dexamethasone-induced repression (Figure 5C).

**Figure 6:**
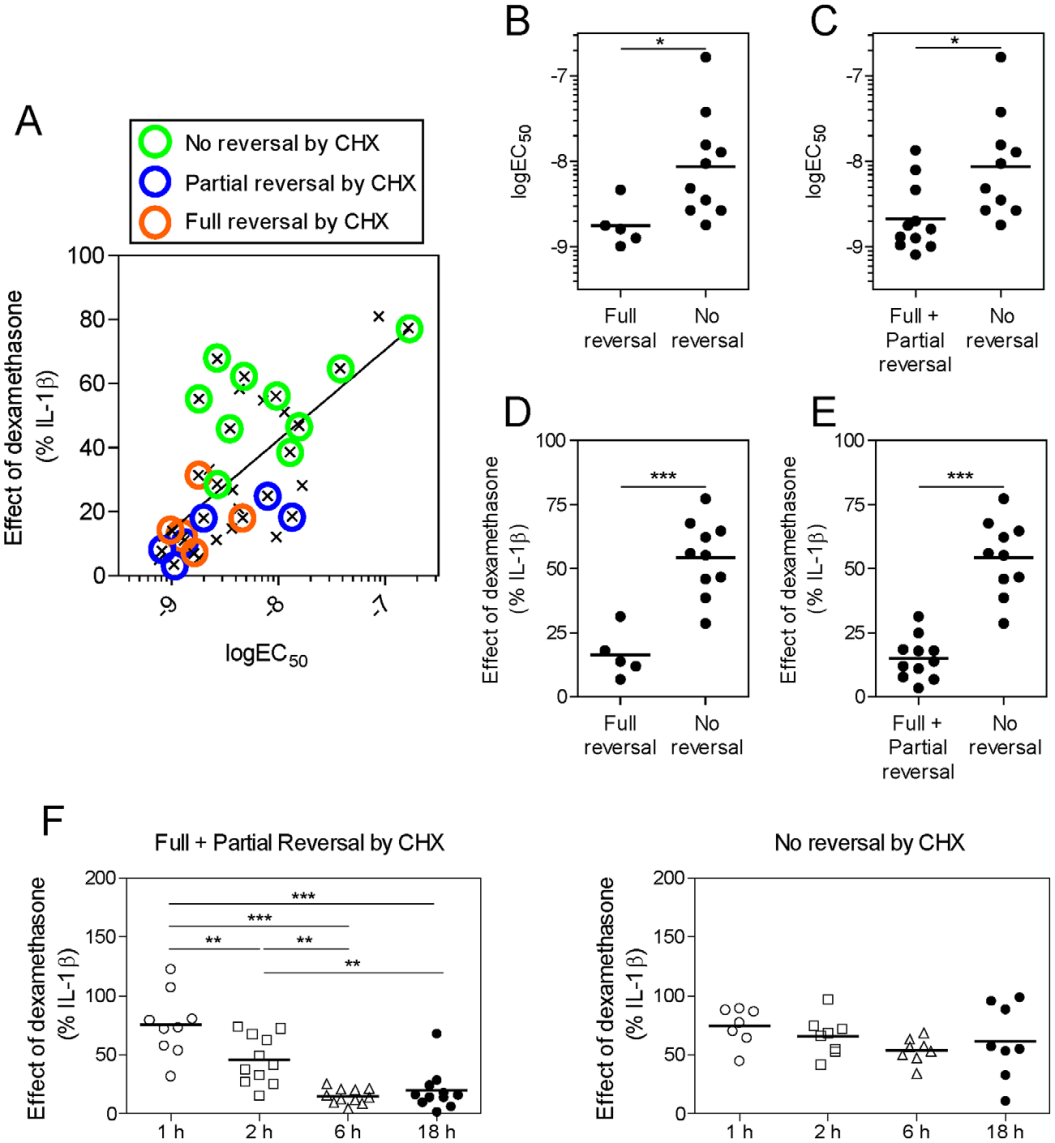
Relationship between the sensitivity and potency of repression by dexamethasone and the reversal by cycloheximide in A549 cells. A. Data showing the effect of 1 µM dexamethasone (as % IL-1β) plotted against the EC_50_ for repression of each target mRNA by dexamethasone (i.e. Figure 2D), were overlaid with the effect of cycloheximide (CHX) on that repression (as shown in Figure 5). The logEC_50_ values for repression by dexamethasone of mRNAs showing: **B,** full reversal, or; **C,** full+partial reversal of this repression by cycloheximide were compared with the logEC_50_ values for mRNAs showing no reversal of dexamethasone-dependent repression by cycloheximide. Likewise, the effect of dexamethasone at 1 µM, expressed as a percentage of IL-1β, was compared for mRNAs showing: **D,** full reversal, or; **E,** full+partial reversal of this repression by cycloheximide were compared with mRNAs showing no reversal of dexamethasone-dependent repression by cycloheximide. Statistical analyses were performed by unpaired t-test. * *P*<0.05, *** *P*<0.001. **F.** Data showing the effect of 1 µM dexamethasone at 1, 2, 6 and 18 h (from Figure 1) are plotted for the group of genes showing reversal of repression by cycloheximide (left graph) and the group of genes showing no reversal (right graph). Statistical analysis was performed using ANOVA with a Bonferroni post-test and is indicated: *, *P*<0.05; **, *P*<0.01; ***, *P*<0.001.

These data indicate that >50% of the IL-1β-induced mRNAs examined in the present study show dexamethasone-repression in manner that requires protein synthesis. This implies a transactivation-dependent mechanism. In contrast, there were 10 IL-1β-induced mRNAs (just under 50%) whose dexamethasone-repression was refractory to the effects of cycloheximide and may therefore correlate with a classical transrepression type mechanism. However, the genes showing cycloheximide reversal of repression also showed high levels of repression by dexamethasone (Figure 5A and 5B). Conversely, the genes with no reversal of dexamethasone-dependent repression by cycloheximide showed lesser levels of dexamethasone-dependent repression (Figure 5C). To explore this further, the data from Figure 2C were overlaid with the effects of cycloheximide (Figure 6A). The genes where cycloheximide reversed (fully or partially) the dexamethasone-dependent repression were clustered in the lower left-hand part of the graph showing that they were the most affected by dexamethasone, in terms of both magnitude of repression (E_max_) and sensitivity to inhibition (EC_50_) (Figure 6A). Conversely, genes for which cycloheximide did not reverse the dexamethasone-dependent repression were inhibited weakly and with low potency by dexamethasone. In each case, the logEC_50_ (Figure 6B & C) and the effect of dexamethasone (% of IL-1β treated) (Figure 6D and 6E) for genes showing full or partial reversal were significantly different from the genes where there was no reversal by cycloheximide. These data therefore indicate two mechanistically distinct processes of repression (cycloheximide-sensitive and -insensitive) in which the most sensitively repressed genes require ongoing gene expression for repression by dexamethasone. Furthermore, examining the repression over time for each group of genes, also suggests different mechanisms of repression (Figure 6F). Thus, the genes for which cycloheximide reversed the dexamethasone-dependent repression showed significantly more repression at longer times of incubation. i.e. The level of repression increased over time (Figure 6F, left graph). Conversely, there was no significant change in the level of repression over time for the genes that were not reversed by cycloheximide i.e. repression was not time-dependent (Figure 6F, right graph). These data suggest that the cycloheximide sensitive repression is time-dependent, whereas the cycloheximide insensitive repression is not, again arguing for two distinct mechanisms of repression.

## Discussion

The current study focuses on mechanisms underlying the ability of the anti-inflammatory glucocorticoid, dexamethasone, to repress the mRNA expression of inflammatory genes induced by IL-1β. Using Affymetrix HG-U95Av2 and B microarrays, just under 430 mRNAs were identified that were induced 2 fold or more by IL-1β (at 6 h). The effects of dexamethasone, in the presence of IL-1β, on these genes was highly variable, with a few genes showing further enhanced expression, many genes showing little effect of dexamethasone and the majority revealing reduced expression (Figure S1 and Table S2). This finding is supported by real-time PCR data showing repression for 34 out of 39 IL-1β-induced mRNAs. While the reversal of this dexamethasone-dependent repression by both a GR antagonist, ORG34517, and GR-specific siRNA clearly supports a role for GR, it is equally clear that such reversals were often only partial. In the case of ORG34517, this could be due to the nature of the effect of ORG34517 on GR. Thus on a low potency response such as the 2×GRE reporter (EC_50_ for dexamethasone = 34 nM), ORG34517 acts as a competitive antagonist (Figure 3A). However, on higher potency responses, such as the repression of IL-1β-induced mRNAs (EC_50_ values for Dex 0.7–10 nM), it is clear that ORG34715 can exert agonistic effects (Table S4). In such instances antagonism of the full agonist (dexamethasone) is limited to the extent of the agonism of the partial agonist (ORG34517) (an effect referred to as competitive dualism). It is also important to note that the GR-specific siRNA does not completely prevent GR expression and this correlates with reduced, but not absent, 2×GRE-dependent transcription in the presence of the GR-specific siRNA (Figures 3B and 3C). Thus, low levels of GR may be sufficient for dexamethasone-dependent repression of inflammatory gene mRNA expression. However, despite these shortcomings, our data indicate that the repression of IL-1β-induced gene expression by dexamethasone is GR-dependent.

The finding that 34 out of 39 IL-1β-induced genes were reduced by dexamethasone raises a critical point: Not all inflammatory genes are repressed by glucocorticoids meaning glucocorticoids show differential effects on inflammatory responses [47]. Indeed, the current data emphasise a need to understand, in detail, the functional significance of those genes that are normally repressed by glucocorticoids, compared to genes that are not repressed. Such analyses will promote an appreciation as to which aspects of inflammation are more or less sensitive to glucocorticoid therapy. Thus, despite immune suppression being an established side-effect, glucocorticoids have been reported to spare some innate immune responses [47]. Equally, asthma is generally well controlled by glucocorticoid therapy, whereas chronic obstructive pulmonary disease, viral exacerbations of asthma or asthmatics who smoke respond poorly to glucocorticoids [2]. While traditionally thought of as showing that these conditions are due to an induced resistance to glucocorticoid, a different view, and one that the current data support, may be that the nature of the inflammatory response is in fact inherently less responsive to glucocorticoid. Clearly, considerable work is needed to investigate this effect, particularly in disease-relevant primary cells. Our PCR analysis shows that IL-1β-induced CSF3, IL32 and TNFAIP3 are all spared the repressive effect of glucocorticoids and these genes play key functional roles in promoting aspects of innate and adaptive immunity. Thus CSF3 is critical for macrophage and neutrophil proliferation and maturation, while IL32 is a core inflammatory cytokine that activates NF-κB and induces expression of multiple inflammatory mediators from multiple cells, including macrophages [48], [49]. Conversely, TNFAIP3, also called A20, plays a key role in feedback regulation of NF-κB and inflammatory responses [50]. Consequently, it is essential that glucocorticoids do not materially down-regulate TNFAIP3 expression as this would have the effect of promoting inflammatory gene expression. Likewise, SOD2, exerts an essential protective function by clearing superoxide radicals that are generated during normal inflammatory processes [51] and was unaffected by treatment with dexamethasone. Equally, genes such as IRF1, a key regulator of adaptive Th1 responses and innate immunity [52], are only modestly repressed by glucocorticoid. Likewise, complement factor B (CFB), an essential factor in the alternative complement cascade, promotes airways hyperreactivity [53], but protects from recurrent infections, shows only partial repression by dexamethasone [54].

The NF-κB signalling pathway is often cited as a primary target of glucocorticoid action. However, many studies report only slight or partial effects of glucocorticoids on NF-κB-dependent transcription [16], [40], [41]. Indeed, in some contexts, the NF-κB and glucocorticoid pathways work together to promote transcription [55], [56], including the expression of anti-apoptotic genes, including c-IAP2 (BIRC3) [57], or chemokines [58]. Such data, along with the known roles of genes such as TNFAIP3 or SOD2, are obviously incompatible with a general repressive effect of glucocorticoids that acts on NF-κB. In the current study, we demonstrate a clear separation between NF-κB as an activator of gene expression and a generic mechanism of glucocorticoid repression. Thus, the five genes that were not repressed by dexamethasone were highly NF-κB-dependent. Other inflammatory genes that revealed only partial repression by glucocorticoid were also highly NF-κB-dependent. At a more generic level, no correlation between the induction by IL-1β and the repression achieved by dexamethasone was noted, also arguing for independent pathways of up-regulation by IL-1β and down-regulation by glucocorticoid.

One aspect of independent regulation, but nevertheless possible interaction between glucocorticoid and inflammatory pathways is the suggestion that some IL-1β-induced genes are also up-regulated by dexamethasone alone. Thus, the microarray identified 3 genes (PRIC285, BIRC3, DOCK4) from those induced 4-fold or more by IL-1β that showed significant up-regulation by dexamethasone. For the 39 IL-1β-induced genes that were analysed by PCR, dexamethasone-induced expression of PRIC285 and BIRC3 was confirmed. In addition, the greater sensitivity of real-time PCR also revealed significant up-regulation of CSF3, G0S2, IFIT1, LAMB3 and TNFAIP3 by dexamethasone. Thus, it is possible that an independent ability of glucocorticoid to induce expression of such genes may help maintain IL-1β-induced expression in the presence of glucocorticoid, which may, in fact, also act to switch off many of the pathways that are activated by IL-1β. Certainly, CSF3, BIRC3, TNFAIP3 and G0S2 are IL-1β-induced genes that were the least affected by dexamethasone. Interestingly, IL32, which also falls into this group, was induced >2-fold by dexamethasone (although this effect did not reach significance). Such data illustrate a mechanism by which certain responses could be maintained in the context of increased glucocorticoids. Indeed, our array data confirms modest up-regulation of NFKBIA by dexamethasone, yet IL-1β-induced expression was unaffected by glucocorticoid, as previously shown [16]. Therefore the modest up-regulation of NFKB1A by glucocorticoids may aid in maintaining its expression, and therefore feedback control of NF-κB. Of note is the fact that we have observed an equivalent effect for the mRNA destabilising protein tristetraprolin (TTP) or ZFP36. This is primarily induced by IL-1β to exert feedback control of inflammatory genes, such as IL-8 and GM-CSF, and is only modestly induced by dexamethasone [59]. Hence we speculate that glucocorticoid-inducibility of a subset of inflammatory genes may help preserve various components of the inflammatory response, for example by maintaining aspects of host defence or necessary feedback regulation. Assuming transcriptional regulation for this modest glucocorticoid-inducibility, such data predict the existence of identifiable glucocorticoid responsive regions in the promoters of these inflammatory genes. Indeed, a study of GR binding sites in dexamethasone responsive genes found that both BIRC3 and TNFAIP3 are bound by GR within 10 kB of the transcription start site, consistent with up-regulation of these genes by dexamethasone in the current study [23].

While real-time PCR analysis allowed the 39 IL-1β-induced mRNAs to be grouped according to expression kinetic (early, intermediate or late), we were not able to identify obvious differences in responsiveness to dexamethasone between these groups. All three groups contained genes whose expression was highly, lowly or partially repressed by dexamethasone in a GR-dependent manner. While, these groupings were merely based on temporal differences in expression, it was predicted that those genes revealing a delayed kinetic of induction (i.e. late phase) would represent genes whose expression depends on some time-dependent event, for example, the prior expression of another factor that was essential for expression. The fact that the protein synthesis inhibitor, cycloheximide, prevented or reduced expression of many of these late genes, yet none of the early genes were affected, supports this view. Equally, cycloheximide significantly blocked the expression of four genes (BCL2A1, G0S2, IL1B and TFF1) in the intermediate group, suggesting that their expression was also dependent on the synthesis of one or more new factors. All such cycloheximide-sensitive genes were necessarily excluded from the later parts of our analysis as the effect of inhibiting proteins synthesis on the repression by dexamethasone cannot be tested on genes whose expression is itself sensitive to protein synthesis inhibition. Thus the main findings of our study are restricted to acute phase genes whose expression induced by IL-1β was not attenuate by blockade of protein synthesis.

Possibly the most surprising and important finding to come out of the current study was the correlation between potency (EC_50_) of repression by dexamethasone with the extent of repression. While variation in these parameters was obvious and intuitive, it was unexpected that a correlation would exist such that IL-1β-induced genes showing the greatest repression by dexamethasone were also the most potently repressed by dexamethasone. This result suggests that in the context of a sub-maximal level of glucocorticoid, such as may occur in patients undergoing inhaled glucocorticoid therapy in the treatment of asthma, these would represent the most sensitive genes to repression by glucocorticoids and may therefore represent the main drivers of the response to therapy. Equally, this implies that mechanisms induced by the glucocorticoid and which cause this repression must also be active at such concentrations of glucocorticoid. If the effects of protein synthesis inhibition are now considered, these data reveal that the IL-1β-induced genes whose expression was repressed by dexamethasone in a cycloheximide-sensitive manner are the genes most repressed and most potently repressed by dexamethasone. Thus, a requirement for ongoing gene expression in these repressive events is implicated and this is consistent with prior studies that implicated the repression of PTGS2, CXCL1, IL6, IL8 and CSF2 as being at least partially dependent on glucocorticoid-induced expression of DUSP1 or TSC22D3 [31], [32], [36], [60]–[62]. Hence, the ability to prevent the repressive effects of dexamethasone with cycloheximide is consistent with repression occurring via the glucocorticoid-dependent expression of repressive genes, i.e. requires transactivation by GR. As such, the induction of DUSP1 occurs with an EC_50_ in the low nanomolar range, a potency similar to the repression of the most sensitively repressed inflammatory genes [41]. Furthermore, many glucocorticoid-induced genes reveal even greater potencies (low EC_50_) and this suggests that repression occurring via the induction of anti-inflammatory genes is highly plausible [23]. As noted above, late phase genes were excluded from this analysis due to their expression being inhibited in the presence of cycloheximide. This meant that the role of glucocorticoid-inducible genes could only be examined in respect of early phase genes. Whether this introduces an unintentional bias into the results of these experiments is unclear. However, it is likely that late phase genes would also be inhibited by a combination of mechanisms, especially considering that there are both genes whose expression is highly or lowly repressed by dexamethasone in this group, as is seen with the early phase group.

Equally, the IL-1β-induced genes which show cycloheximide-dependent repression are those genes for which a strong time-dependence to their repression is observed (Figure 6F). This suggests a need for time-dependent processes, such as new gene expression, to occur prior to the onset of repression. Conversely, genes not showing a cycloheximide-dependent component to the repression by dexamethasone are consistent with a conventional transrepressive mechanism. This mechanism does not involve a gene expression-dependent event, but rather is reported to occur via a direct interaction with GR and the recruitment of HDAC activity to targeted promoters [63]. Interestingly, this group of genes does not display time-dependent repression (Figure 6F) and is consistent with a rapid onset of repression due to GR recruitment. Finally, it is important to realise that a number of genes revealed both cycloheximide-sensitive and -insensitive components to their repression by dexamethasone. This suggests that these inflammatory genes are subject to repression via both GR transactivation and transrepression. Given recent findings that implicate the glucocorticoid induction of DUSP1 in the repression of both AP-1 and NF-κB [41], [64], both targets of classical transrepression [5], it is perhaps not surprising that at least some mRNAs show glucocorticoid-dependent repression that involves both classical GR transrepression and transactivation. Thus, there exists considerable possibility for redundancy both between and within these two mechanisms of repression. In this regard, multiple mechanisms of transrepression are established [5]. Likewise, NFKB1A, TSC22D3 and DUSP1 and others are up-regulated by glucocorticoids and may all impact on pathways involved in inflammatory gene expression [27]. Moreover, cycloheximide would not prevent the effect of induced RNAs, for example microRNA (miRNA), in the repression of inflammatory gene expression. It is therefore possible that glucocorticoids, by inducing miRNA expression, could then inhibit inflammatory gene expression.

In conclusion, our data show that a single, global mechanism of inflammatory gene repression does not account for all of the anti-inflammatory actions of glucocorticoids. Indeed, more gene-specific methods of inhibition that could involve transrepressive and/or transactivation mechanisms of repression are indicated. Importantly, the inhibition of inflammatory genes that are highly repressed by glucocorticoids appears to involve the induction of anti-inflammatory gene expression and occurs at low concentrations of glucocorticoid. These data highlight the importance of GR-induced transactivation for the effective repression of inflammatory gene expression by glucocorticoids.

## Supporting Information

Figure S1 **Microarray analysis of the effect of IL-1β and dexamethasone on A549 cells.** A549 cells were either not stimulated or treated with IL-1β (1 ng/ml), dexamethasone (Dex) (1 µM) or a combination of the two for 6 or 18 h (n = 3). RNA was extracted and microarray profiling conducted using human genome U95Av2 and B GeneChip expression arrays. (**A**) Heat map representation of all genes induced 4 fold or more by IL-1β at 6 h. The complete, unsorted microarray dataset at 6 and 18 h was sorted based on IL-1β induction at 6 h and all rows with less than 2 fold inducibility were removed. In addition, any rows where there was no “present” call (p) (indicating presence of a transcript) at either 6 or 18 h was removed. Datasets with the same gene name were merged to give average fold inductions and the data was then sorted based on fold-induction by IL-1β at 6 h. Heat map is colour coded based on fold induction values, as indicated in the legend. (**B**) Heat map representation of all genes analysed in the current study. Fold induction values for dexamethasone (Dex), IL-1β or the combination are indicated. (**C**) Effect of dexamethasone (Dex) as a fold of IL-1β (i.e. IL-1β = 1) for all genes induced 4 fold or more by IL-1β (from A). Genes are divided into 5 groups based on fold induction by dexamethasone (as indicated at the bottom of each graph): Group 1, ≥1.25 fold; Group 2, ≥0.75 but <1.25 fold; Group 3, ≥0.5 but <0.75 fold; Group 4, ≥0.25 but <0.5 fold; Group 5, <0.25 fold. (**D**) Effect of dexamethasone as a percentage of IL-1β is plotted against fold induction for each gene. Linear regression was performed using GraphPad Prizm software.
(PDF)

Figure S2 **Timecourse analysis of inflammatory mRNA expression.** A549 cells were either not stimulated (NS) or treated with IL-1β (1 ng/ml), dexamethasone (Dex) (1 µM) or a combination of the two for 1, 2, 6 or 18 h. Cells were then harvested for RNA and real-time PCR was carried out for the indicated mRNAs and GAPDH. Data (n = 3) normalised to GAPDH and expressed as fold over NS at 1 h are plotted as mean ± SE. Genes are grouped based on expression patterns: (**A**) ‘Early-phase’ genes are those which have a peak of expression at 1 or 2 h; (**B**) ‘Intermediate’ genes have a peak of expression at 2, 6 or 18 h; (**C**) ‘Late-phase’ genes have a peak of expression at 6 h or later with less than 50% of that peak expression observed at 1 or 2 h. Significance relative to IL-1β treated samples at each time point was tested using ANOVA with a Bonferroni post-test and is indicated: *, *P*<0.05; **, P<0.01; ***, P<0.001.
(PDF)

Figure S3 **Effect of increasing concentrations of dexamethasone on IL-1β-induced inflammatory gene expression.** A549 cells were either not stimulated (NS) or treated with IL-1β (1 ng/ml) and increasing concentrations of dexamethasone (as indicated) for 6 h prior to harvesting for RNA. Real-time PCR was carried out for GAPDH and the indicated genes. Data (n = 6) normalised to GAPDH and expressed as percentage of IL-1β are plotted as mean ± S.E. Significance relative to IL-1β-treated samples using ANOVA with a Dunnett’s post-test is indicated: *, *P*<0.05; **, P<0.01; ***, P<0.001.
(PDF)

Figure S4 **Relationship between the effect of dexamethasone and Ad5-IκBαΔN.** The effect of dexamethasone (1 µM) is plotted against the effect of Ad5-IκBαΔN (MOI 100), each following IL-1β treatment for 6 h and both expressed as percentage of IL-1β, for each mRNA. Data are derived from Figure 2A and 4C respectively. Linear regression was performed using GraphPad Prizm software.
(PDF)

Figure S5 **Effect of cycloheximide on IL-1β-induced inflammatory mRNA expression.** A549 cells were treated with IL-1β (1 ng/ml) in the absence or presence of cycloheximide (CHX) (100 µg/ml) for 4 h. Cells were then harvested for real-time PCR analysis of the indicated genes and GAPDH. Data (n = 4) normalised to GAPDH and expressed as percentage of IL-1β treated samples are plotted as means ± SE. Significant repression relative to IL-1β treated samples was tested using a paired, one-way t-test and is indicated: *, *P*<0.05; **, P<0.01; ***, P<0.001.
(PDF)

Table S1 **Primer sequences used in the study.** Forward (F) and reverse (R) primer sequences (5′-3′) are shown in addition to the accession number for each gene. For genes with more than one RefSeq splice variant, primers were designed to amplify all variants, with the exception of IFIT3 for which two sets of primers were designed.
(DOCX)

Table S2 **Microarray analysis of the effect of IL-1β and dexamethasone on gene expression in A549 cells.** Table of microarray data for all genes induced 2 fold or more by IL-1β at 6 h. A549 cells were either not treated or stimulated with IL-1β (1 ng/ml), dexamethasone (Dex) (1 µM) or a combination of the two for 6 or 18 h (n = 3). RNA was extracted and microarray profiling conducted using human genome U95Av2 and B GeneChip expression arrays. The complete, unsorted microarray dataset at 6 and 18 h was sorted based on IL-1β induction at 6 h and all rows with less than 2 fold inducibility were removed. In addition, any rows where there was no p value (indicating presence of a transcript) at either 6 or 18 h was removed. Datasets with the same gene name were merged to give average fold inductions and the data was then sorted based on fold-induction by IL-1β at 6 h. Data (n = 3) are expressed as fold over non-stimulated samples. P values indicating level of significance are given relative to non-stimulated values unless otherwise indicated. The datasets are divided into two: **A.** represents all probesets that correlate with a known gene locus as indicated by gene name in column 1 whereas **B.** represents all probe-sets for which there is no known gene associated.
(XLSX)

Table S3 **Effect of dexamethasone on inflammatory mRNA expression.** The effect of dexamethasone (Dex) (1 µM) is shown on the induction of inflammatory mRNAs by IL-1β (1 ng/ml) at 6 h. The effect of dexamethasone is expressed as percentage of IL-1β treated. Data are derived from Figure 2A (Effect of Dex) and Figure S3 (EC_50_). There is no EC_50_ value available (n/a) for mRNAs that were not significantly repressed by dexamethasone.
(DOCX)

Table S4 **Effect of ORG34517 on IL-1β-induced gene expression and effect of lamin siRNA on dexamethasone-dependent repression of inflammatory mRNA expression. A.** Effect of ORG34517 on inflammatory gene expression. A549 cells were either not stimulated or incubated with ORG34517 for 30 min prior to stimulation with IL-1β (1 ng/ml) for 6 h. RNA was extracted and real-time PCR analysis carried out for the indicated mRNAs. Data (n = 4–6) are expressed as percentage of IL-1β-treated samples. Statistical analysis was performed by paired t-test: *, *P*<0.05. **B.** Effect of lamin (control siRNA) on repression of inflammatory mRNA expression by dexamethasone. A549 cells were incubated with lamin siRNA for 24 h prior to stimulation with IL-1β (1 ng/ml) for 6 h in the absence or presence of dexamethasone (Dex) (1 or 0.1 µM). Data (n = 5) are expressed as percentage of IL-1β. Significance, relative to IL-1β+Dex was tested using ANOVA with a Dunnett’s post-test (see Figure 3D for other half of this analysis: IL-1β+Dex+lamin siRNA vs IL-1β+Dex+GR siRNA). ***, *P*<0.001.
(DOCX)

Materials and Methods S1 **Additional materials and methods.**
(DOCX)

## References

[1] Barnes PJ (2006) Corticosteroids: the drugs to beat. Eur J Pharmacol 533: 2–14. 16436275 10.1016/j.ejphar.2005.12.052

[2] Newton R, Leigh R, Giembycz MA (2010) Pharmacological strategies for improving the efficacy and therapeutic ratio of glucocorticoids in inflammatory lung diseases. Pharmacol Ther 125: 286–327. 19932713 10.1016/j.pharmthera.2009.11.003

[3] Newton R (2000) Molecular mechanisms of glucocorticoid action: what is important? Thorax 55: 603–613. 10856322 10.1136/thorax.55.7.603 PMC1745805

[4] Rhen T, Cidlowski JA (2005) Antiinflammatory action of glucocorticoids–new mechanisms for old drugs. N Engl J Med 353: 1711–1723. 16236742 10.1056/NEJMra050541

[5] De Bosscher K, Vanden Berghe W, Haegeman G (2003) The interplay between the glucocorticoid receptor and nuclear factor-kappaB or activator protein-1: molecular mechanisms for gene repression. Endocr Rev 24: 488–522. 12920152 10.1210/er.2002-0006

[6] Clark AR, Belvisi MG (2012) Maps and legends: the quest for dissociated ligands of the glucocorticoid receptor. Pharmacol Ther 134: 54–67. 22212616 10.1016/j.pharmthera.2011.12.004

[7] Ito K, Barnes PJ, Adcock IM (2000) Glucocorticoid receptor recruitment of histone deacetylase 2 inhibits interleukin-1beta-induced histone H4 acetylation on lysines 8 and 12. Mol Cell Biol 20: 6891–6903. 10958685 10.1128/mcb.20.18.6891-6903.2000 PMC88765

[8] Barnes PJ (2011) Glucocorticosteroids: current and future directions. Br J Pharmacol 163: 29–43. 21198556 10.1111/j.1476-5381.2010.01199.x PMC3085866

[9] Ray A, Prefontaine KE (1994) Physical association and functional antagonism between the p65 subunit of transcription factor NF-kappa B and the glucocorticoid receptor. Proc Natl Acad Sci U S A 91: 752–756. 8290595 10.1073/pnas.91.2.752 PMC43027

[10] Nissen RM, Yamamoto KR (2000) The glucocorticoid receptor inhibits NFkappaB by interfering with serine-2 phosphorylation of the RNA polymerase II carboxy-terminal domain. Genes Dev 14: 2314–2329. 10995388 10.1101/gad.827900 PMC316928

[11] Luecke HF, Yamamoto KR (2005) The glucocorticoid receptor blocks P-TEFb recruitment by NFkappaB to effect promoter-specific transcriptional repression. Genes Dev 19: 1116–1127. 15879558 10.1101/gad.1297105 PMC1091745

[12] Auphan N, DiDonato JA, Rosette C, Helmberg A, Karin M (1995) Immunosuppression by glucocorticoids: inhibition of NF-kappa B activity through induction of I kappa B synthesis. Science 270: 286–290. 7569976 10.1126/science.270.5234.286

[13] Scheinman RI, Cogswell PC, Lofquist AK, Baldwin AS Jr (1995) Role of transcriptional activation of I kappa B alpha in mediation of immunosuppression by glucocorticoids. Science 270: 283–286. 7569975 10.1126/science.270.5234.283

[14] Brostjan C, Anrather J, Csizmadia V, Stroka D, Soares M, et al (1996) Glucocorticoid-mediated repression of NFkappaB activity in endothelial cells does not involve induction of IkappaBalpha synthesis. J Biol Chem 271: 19612–19616. 8702657 10.1074/jbc.271.32.19612

[15] Ray KP, Farrow S, Daly M, Talabot F, Searle N (1997) Induction of the E-selectin promoter by interleukin 1 and tumour necrosis factor alpha, and inhibition by glucocorticoids. Biochem J 328: 707–715. 9371735 10.1042/bj3280707 PMC1218975

[16] Newton R, Hart LA, Stevens DA, Bergmann M, Donnelly LE, et al (1998) Effect of dexamethasone on interleukin-1beta-(IL-1beta)-induced nuclear factor-kappaB (NF-kappaB) and kappaB-dependent transcription in epithelial cells. Eur J Biochem 254: 81–89. 9652398 10.1046/j.1432-1327.1998.2540081.x

[17] Heck S, Bender K, Kullmann M, Gottlicher M, Herrlich P, et al (1997) I kappaB alpha-independent downregulation of NF-kappaB activity by glucocorticoid receptor. EMBO J 16: 4698–4707. 9303314 10.1093/emboj/16.15.4698 PMC1170096

[18] Sakai DD, Helms S, Carlstedt-Duke J, Gustafsson JA, Rottman FM, et al (1988) Hormone-mediated repression: a negative glucocorticoid response element from the bovine prolactin gene. Genes Dev 2: 1144–1154. 3192076 10.1101/gad.2.9.1144

[19] Drouin J, Trifiro MA, Plante RK, Nemer M, Eriksson P, et al (1989) Glucocorticoid receptor binding to a specific DNA sequence is required for hormone-dependent repression of pro-opiomelanocortin gene transcription. Mol Cell Biol 9: 5305–5314. 2586521 10.1128/mcb.9.12.5305-5314.1989 PMC363695

[20] Bilodeau S, Vallette-Kasic S, Gauthier Y, Figarella-Branger D, Brue T, et al (2006) Role of Brg1 and HDAC2 in GR trans-repression of the pituitary POMC gene and misexpression in Cushing disease. Genes Dev 20: 2871–2886. 17043312 10.1101/gad.1444606 PMC1619949

[21] Wang JC, Derynck MK, Nonaka DF, Khodabakhsh DB, Haqq C, et al (2004) Chromatin immunoprecipitation (ChIP) scanning identifies primary glucocorticoid receptor target genes. Proc Natl Acad Sci U S A 101: 15603–15608. 15501915 10.1073/pnas.0407008101 PMC524211

[22] So AY, Cooper SB, Feldman BJ, Manuchehri M, Yamamoto KR (2008) Conservation analysis predicts in vivo occupancy of glucocorticoid receptor-binding sequences at glucocorticoid-induced genes. Proc Natl Acad Sci U S A 105: 5745–5749. 18408151 10.1073/pnas.0801551105 PMC2311370

[23] Reddy TE, Pauli F, Sprouse RO, Neff NF, Newberry KM, et al (2009) Genomic determination of the glucocorticoid response reveals unexpected mechanisms of gene regulation. Genome Res 19: 2163–2171. 19801529 10.1101/gr.097022.109 PMC2792167

[24] Surjit M, Ganti KP, Mukherji A, Ye T, Hua G, et al (2011) Widespread negative response elements mediate direct repression by agonist-liganded glucocorticoid receptor. Cell 145: 224–241. 21496643 10.1016/j.cell.2011.03.027

[25] Stellato C (2004) Post-transcriptional and nongenomic effects of glucocorticoids. Proc Am Thorac Soc 1: 255–263. 16113443 10.1513/pats.200402-015MS

[26] Clark AR (2007) Anti-inflammatory functions of glucocorticoid-induced genes. Mol Cell Endocrinol 275: 79–97. 17561338 10.1016/j.mce.2007.04.013

[27] Newton R, Holden NS (2007) Separating transrepression and transactivation: a distressing divorce for the glucocorticoid receptor? Mol Pharmacol 72: 799–809. 17622575 10.1124/mol.107.038794

[28] Clark AR, Martins JR, Tchen CR (2008) Role of dual specificity phosphatases in biological responses to glucocorticoids. J Biol Chem 283: 25765–25769. 18541529 10.1074/jbc.R700053200 PMC3258850

[29] Ayroldi E, Riccardi C (2009) Glucocorticoid-induced leucine zipper (GILZ): a new important mediator of glucocorticoid action. FASEB J 23: 3649–3658. 19567371 10.1096/fj.09-134684

[30] Kassel O, Sancono A, Kratzschmar J, Kreft B, Stassen M, et al (2001) Glucocorticoids inhibit MAP kinase via increased expression and decreased degradation of MKP-1. EMBO J 20: 7108–7116. 11742987 10.1093/emboj/20.24.7108 PMC125780

[31] Lasa M, Abraham SM, Boucheron C, Saklatvala J, Clark AR (2002) Dexamethasone causes sustained expression of mitogen-activated protein kinase (MAPK) phosphatase 1 and phosphatase-mediated inhibition of MAPK p38. Mol Cell Biol 22: 7802–7811. 12391149 10.1128/MCB.22.22.7802-7811.2002 PMC134716

[32] Issa R, Xie S, Khorasani N, Sukkar M, Adcock IM, et al (2007) Corticosteroid Inhibition of Growth-Related Oncogene Protein-{alpha} via Mitogen-Activated Kinase Phosphatase-1 in Airway Smooth Muscle Cells. J Immunol 178: 7366–7375. 17513787 10.4049/jimmunol.178.11.7366

[33] Kelly M, King E, Rider C, Gwozd C, Holden N, et al.. (2011) Corticosteroid-induced gene expression in allergen-challenged asthmatic subjects taking inhaled budesonide. Br J Pharmacol. 10.1111/j.1476-5381.2011.01620.x PMC3372826 21827450

[34] Mittelstadt PR, Ashwell JD (2001) Inhibition of AP-1 by the glucocorticoid-inducible protein GILZ. J Biol Chem 276: 29603–29610. 11397794 10.1074/jbc.M101522200

[35] Ayroldi E, Migliorati G, Bruscoli S, Marchetti C, Zollo O, et al (2001) Modulation of T-cell activation by the glucocorticoid-induced leucine zipper factor via inhibition of nuclear factor kappaB. Blood 98: 743–753. 11468175 10.1182/blood.v98.3.743

[36] Eddleston J, Herschbach J, Wagelie-Steffen AL, Christiansen SC, Zuraw BL (2007) The anti-inflammatory effect of glucocorticoids is mediated by glucocorticoid-induced leucine zipper in epithelial cells. J Allergy Clin Immunol 119: 115–122. 17208592 10.1016/j.jaci.2006.08.027

[37] Ayroldi E, Zollo O, Bastianelli A, Marchetti C, Agostini M, et al (2007) GILZ mediates the antiproliferative activity of glucocorticoids by negative regulation of Ras signaling. J Clin Invest 117: 1605–1615. 17492054 10.1172/JCI30724 PMC1865030

[38] Kwon OJ, Au BT, Collins PD, Baraniuk JN, Adcock IM, et al (1994) Inhibition of interleukin-8 expression by dexamethasone in human cultured airway epithelial cells. Immunology 81: 389–394. 8206512 PMC1422337

[39] Newton R, Eddleston J, Haddad E, Hawisa S, Mak J, et al (2002) Regulation of kinin receptors in airway epithelial cells by inflammatory cytokines and dexamethasone. Eur J Pharmacol 441: 193–202. 12063092 10.1016/s0014-2999(01)01624-7

[40] Catley MC, Sukkar MB, Chung KF, Jaffee B, Liao SM, et al (2006) Validation of the anti-inflammatory properties of small-molecule IkappaB Kinase (IKK)-2 inhibitors by comparison with adenoviral-mediated delivery of dominant-negative IKK1 and IKK2 in human airways smooth muscle. Mol Pharmacol 70: 697–705. 16687566 10.1124/mol.106.023150

[41] King EM, Holden NS, Gong W, Rider CF, Newton R (2009) Inhibition of NF-kappaB-dependent transcription by MKP-1: transcriptional repression by glucocorticoids occurring via p38 MAPK. J Biol Chem 284: 26803–26815. 19648110 10.1074/jbc.M109.028381 PMC2785369

[42] Chivers JE, Gong W, King EM, Seybold J, Mak JC, et al (2006) Analysis of the dissociated steroid, RU24858, does not exclude a role for inducible genes in the anti-inflammatory actions of glucocorticoids. Mol Pharmacol 70: 2084–2095. 16988013 10.1124/mol.106.025841

[43] Kaur M, Chivers JE, Giembycz MA, Newton R (2008) Long-acting beta2-adrenoceptor agonists synergistically enhance glucocorticoid-dependent transcription in human airway epithelial and smooth muscle cells. Mol Pharmacol 73: 203–214. 17901197 10.1124/mol.107.040121

[44] Peeters BW, Ruigt GS, Craighead M, Kitchener P (2008) Differential effects of the new glucocorticoid receptor antagonist ORG 34517 and RU486 (mifepristone) on glucocorticoid receptor nuclear translocation in the AtT20 cell line. Ann N Y Acad Sci 1148: 536–541. 19120154 10.1196/annals.1410.072

[45] Holden NS, Gong W, King EM, Kaur M, Giembycz MA, et al (2007) Potentiation of NF-kappaB-dependent transcription and inflammatory mediator release by histamine in human airway epithelial cells. Br J Pharmacol 152: 891–902. 17891168 10.1038/sj.bjp.0707457 PMC2078227

[46] Catley MC, Chivers JE, Holden NS, Barnes PJ, Newton R (2005) Validation of IKK beta as therapeutic target in airway inflammatory disease by adenoviral-mediated delivery of dominant-negative IKK beta to pulmonary epithelial cells. Br J Pharmacol 145: 114–122. 15723090 10.1038/sj.bjp.0706170 PMC1576123

[47] Schleimer RP (2004) Glucocorticoids suppress inflammation but spare innate immune responses in airway epithelium. Proc Am Thorac Soc 1: 222–230. 16113438 10.1513/pats.200402-018MS

[48] Cornish AL, Campbell IK, McKenzie BS, Chatfield S, Wicks IP (2009) G-CSF and GM-CSF as therapeutic targets in rheumatoid arthritis. Nat Rev Rheumatol 5: 554–559. 19798030 10.1038/nrrheum.2009.178

[49] Commins SP, Borish L, Steinke JW (2010) Immunologic messenger molecules: cytokines, interferons, and chemokines. J Allergy Clin Immunol 125: S53–S72. 19932918 10.1016/j.jaci.2009.07.008

[50] Vereecke L, Beyaert R, van Loo G (2011) Genetic relationships between A20/TNFAIP3, chronic inflammation and autoimmune disease. Biochem Soc Trans 39: 1086–1091. 21787353 10.1042/BST0391086

[51] Fukai T, Ushio-Fukai M (2011) Superoxide dismutases: role in redox signaling, vascular function, and diseases. Antioxid Redox Signal 15: 1583–1606. 21473702 10.1089/ars.2011.3999 PMC3151424

[52] Honda K, Taniguchi T (2006) IRFs: master regulators of signalling by Toll-like receptors and cytosolic pattern-recognition receptors. Nat Rev Immunol 6: 644–658. 16932750 10.1038/nri1900

[53] Taube C, Thurman JM, Takeda K, Joetham A, Miyahara N, et al (2006) Factor B of the alternative complement pathway regulates development of airway hyperresponsiveness and inflammation. Proc Natl Acad Sci U S A 103: 8084–8089. 16702544 10.1073/pnas.0602357103 PMC1472433

[54] Bartholomew WR, Shanahan TC (1990) Complement components and receptors: deficiencies and disease associations. Immunol Ser 52: 33–51. 2091785

[55] Wang Y, Zhang JJ, Dai W, Lei KY, Pike JW (1997) Dexamethasone potently enhances phorbol ester-induced IL-1beta gene expression and nuclear factor NF-kappaB activation. J Immunol 159: 534–537. 9218566

[56] Hofmann TG, Schmitz ML (2002) The promoter context determines mutual repression or synergism between NF-kappaB and the glucocorticoid receptor. Biol Chem 383: 1947–1951. 12553732 10.1515/BC.2002.219

[57] Webster JC, Huber RM, Hanson RL, Collier PM, Haws TF, et al (2002) Dexamethasone and tumor necrosis factor-alpha act together to induce the cellular inhibitor of apoptosis-2 gene and prevent apoptosis in a variety of cell types. Endocrinology 143: 3866–3874. 12239098 10.1210/en.2002-220188

[58] Sukkar MB, Issa R, Xie S, Oltmanns U, Newton R, et al (2004) Fractalkine/CX3CL1 production by human airway smooth muscle cells: induction by IFN-gamma and TNF-alpha and regulation by TGF-beta and corticosteroids. Am J Physiol Lung Cell Mol Physiol 287: L1230–L1240. 15321787 10.1152/ajplung.00014.2004

[59] King EM, Kaur M, Gong W, Rider CF, Holden NS, et al (2009) Regulation of tristetraprolin expression by interleukin-1beta and dexamethasone in human pulmonary epithelial cells: roles for nuclear factor-kappaB and p38 mitogen-activated protein kinase. J Pharmacol Exp Ther 330: 575–585. 19435930 10.1124/jpet.109.151423

[60] Quante T, Ng YC, Ramsay EE, Henness S, Allen JC, et al (2008) Corticosteroids reduce IL-6 in ASM cells via up-regulation of MKP-1. Am J Respir Cell Mol Biol 39: 208–217. 18314542 10.1165/rcmb.2007-0014OC

[61] Turpeinen T, Nieminen R, Moilanen E, Korhonen R (2010) Mitogen-activated protein kinase phosphatase-1 negatively regulates the expression of interleukin-6, interleukin-8, and cyclooxygenase-2 in A549 human lung epithelial cells. J Pharmacol Exp Ther 333: 310–318. 20089808 10.1124/jpet.109.157438

[62] Newton R, King EM, Gong W, Rider CF, Staples KJ, et al (2010) Glucocorticoids inhibit IL-1beta-induced GM-CSF expression at multiple levels: roles for the ERK pathway and repression by MKP-1. Biochem J 427: 113–124. 20100175 10.1042/BJ20091038

[63] Ito K, Yamamura S, Essilfie-Quaye S, Cosio B, Ito M, et al (2006) Histone deacetylase 2-mediated deacetylation of the glucocorticoid receptor enables NF-kappaB suppression. J Exp Med 203: 7–13. 16380507 10.1084/jem.20050466 PMC2118081

[64] Diefenbacher M, Sekula S, Heilbock C, Maier JV, Litfin M, et al (2008) Restriction to Fos family members of Trip6-dependent coactivation and glucocorticoid receptor-dependent trans-repression of activator protein-1. Mol Endocrinol 22: 1767–1780. 18535250 10.1210/me.2007-0574 PMC2505324

