# Supplementary material for: Glucocorticoid Repression of Inflammatory Gene Expression Shows Differential Responsiveness by Transactivation- and Transrepression-Dependent Mechanisms
Source: PLoS One. 2013 Jan 14;8(1):e53936. doi: 10.1371/journal.pone.0053936 (PMC3545719; doi:10.1371/journal.pone.0053936)
Supplement: Figure S1 — Microarray analysis of the effect of IL-1β and dexamethasone on A549 cells. A549 cells were either not stimulated or treated with IL-1β (1 ng/ml), dexamethasone (Dex) (1 µM) or a combination of the two for 6 or 18 h (n = 3). RNA was extracted and microarray profiling conducted using human genome U95Av2 and B GeneChip expression arrays. (A) Heat map representation of all genes induced 4 fold or more by IL-1β at 6 h. The complete, unsorted microarray dataset at 6 and 18 h was sorted based on IL-1β induction at 6 h and all rows with less than 2 fold inducibility were removed. In addition, any rows where there was no “present” call (p) (indicating presence of a transcript) at either 6 or 18 h was removed. Datasets with the same gene name were merged to give average fold inductions and the data was then sorted based on fold-induction by IL-1β at 6 h. Heat map is colour coded based on fold induction values, as indicated in the legend. (B) Heat map representation of all genes analysed in the current study. Fold induction values for dexamethasone (Dex), IL-1β or the combination are indicated. (C) Effect of dexamethasone (Dex) as a fold of IL-1β (i.e. IL-1β = 1) for all genes induced 4 fold or more by IL-1β (from A). Genes are divided into 5 groups based on fold induction by dexamethasone (as indicated at the bottom of each graph): Group 1, ≥1.25 fold; Group 2, ≥0.75 but <1.25 fold; Group 3, ≥0.5 but <0.75 fold; Group 4, ≥0.25 but <0.5 fold; Group 5, <0.25 fold. (D) Effect of dexamethasone as a percentage of IL-1β is plotted against fold induction for each gene. Linear regression was performed using GraphPad Prizm software. (PDF) [file pone.0053936.s001.pdf]

Supporting Figure S1

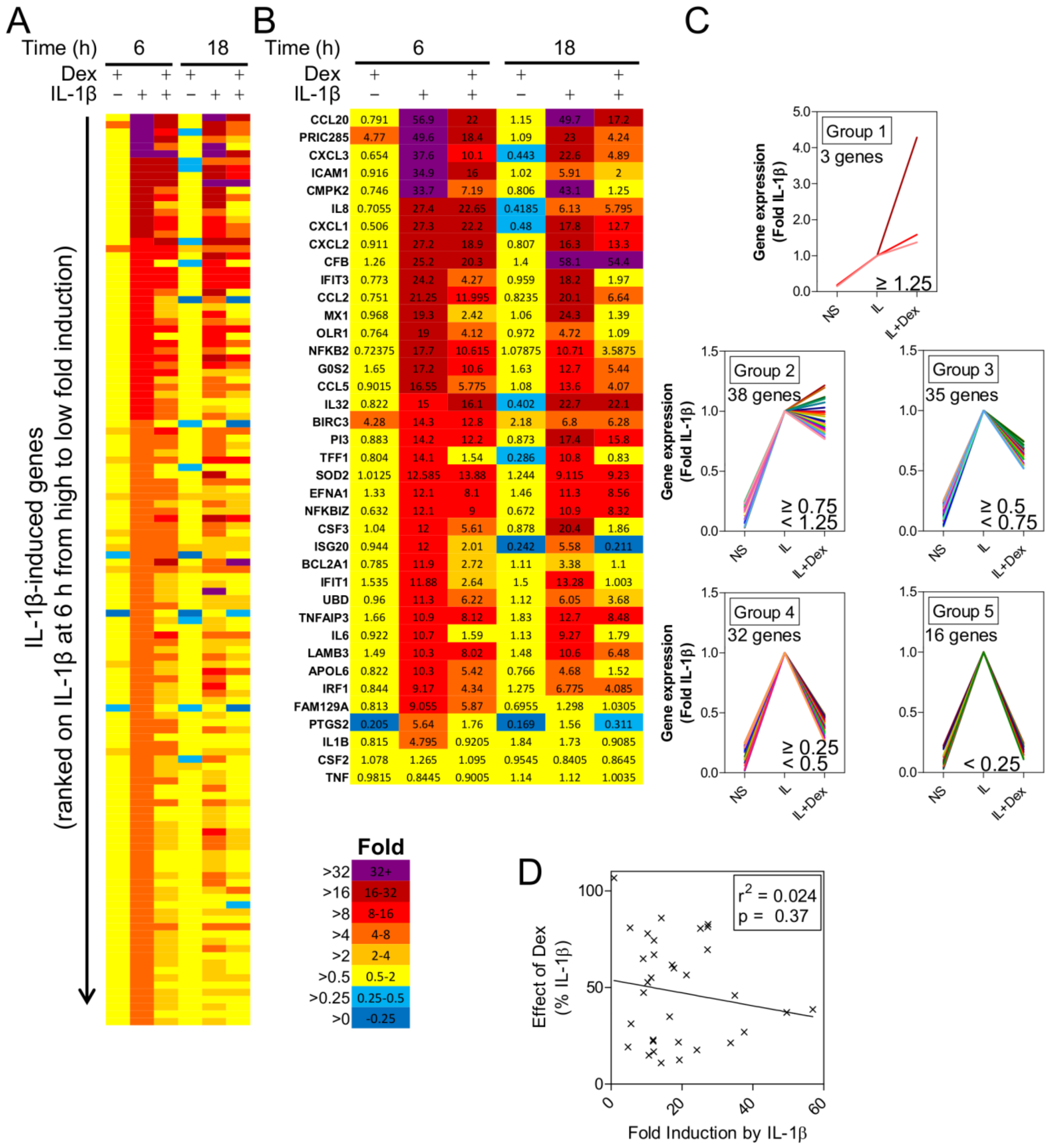

Supporting Figure S1. Microarray analysis of the effect of IL-1β and dexamethasone on A549 cells.

A549 cells were either not stimulated or treated with IL-1β (1 ng/ml), dexamethasone (Dex) (1 µM) or a combination of the two for 6 or 18 h (n = 3). RNA was extracted and microarray profiling conducted using human genome U95Av2 and B GeneChip expression arrays. (**A**) Heat map representation of all genes induced 4 fold or more by IL-1β at 6 h. The complete, unsorted microarray dataset at 6 and 18 h was sorted based on IL-1β induction at 6 h and all rows with less than 2 fold inducibility were

removed. In addition, any rows where there was no "present" call (p) (indicating presence of a transcript) at either 6 or 18 h was removed. Datasets with the same gene name were merged to give average fold inductions and the data was then sorted based on fold-induction by IL-1β at 6 h. Heat map is colour coded based on fold induction values, as indicated in the legend. (**B**) Heat map representation of all genes analysed in the current study. Fold induction values for dexamethasone (Dex), IL-1β or the combination are indicated. (**C**) Effect of dexamethasone (Dex) as a fold of IL-1β (i.e. IL-1β = 1) for all genes induced 4 fold or more by IL-1β (from A). Genes are divided into 5 groups based on fold induction by dexamethasone (as indicated at the bottom of each graph): Group 1, ≥ 1.25 fold; Group 2, ≥ 0.75 but < 1.25 fold; Group 3, ≥ 0.5 but < 0.75 fold; Group 4, ≥ 0.25 but < 0.5 fold; Group 5, < 0.25 fold. (**D**) Effect of dexamethasone as a percentage of IL-1β is plotted against fold induction for each gene. Linear regression was performed using GraphPad Prizm software.
