# Supplementary material for: Glucocorticoid Repression of Inflammatory Gene Expression Shows Differential Responsiveness by Transactivation- and Transrepression-Dependent Mechanisms
Source: PLoS One. 2013 Jan 14;8(1):e53936. doi: 10.1371/journal.pone.0053936 (PMC3545719; doi:10.1371/journal.pone.0053936)
Supplement: Figure S2 — Timecourse analysis of inflammatory mRNA expression. A549 cells were either not stimulated (NS) or treated with IL-1β (1 ng/ml), dexamethasone (Dex) (1 µM) or a combination of the two for 1, 2, 6 or 18 h. Cells were then harvested for RNA and real-time PCR was carried out for the indicated mRNAs and GAPDH. Data (n = 3) normalised to GAPDH and expressed as fold over NS at 1 h are plotted as mean ± SE. Genes are grouped based on expression patterns: (A) ‘Early-phase’ genes are those which have a peak of expression at 1 or 2 h; (B) ‘Intermediate’ genes have a peak of expression at 2, 6 or 18 h; (C) ‘Late-phase’ genes have a peak of expression at 6 h or later with less than 50% of that peak expression observed at 1 or 2 h. Significance relative to IL-1β treated samples at each time point was tested using ANOVA with a Bonferroni post-test and is indicated: *, P<0.05; **, P<0.01; ***, P<0.001. (PDF) [file pone.0053936.s002.pdf]

Supporting Figure S2

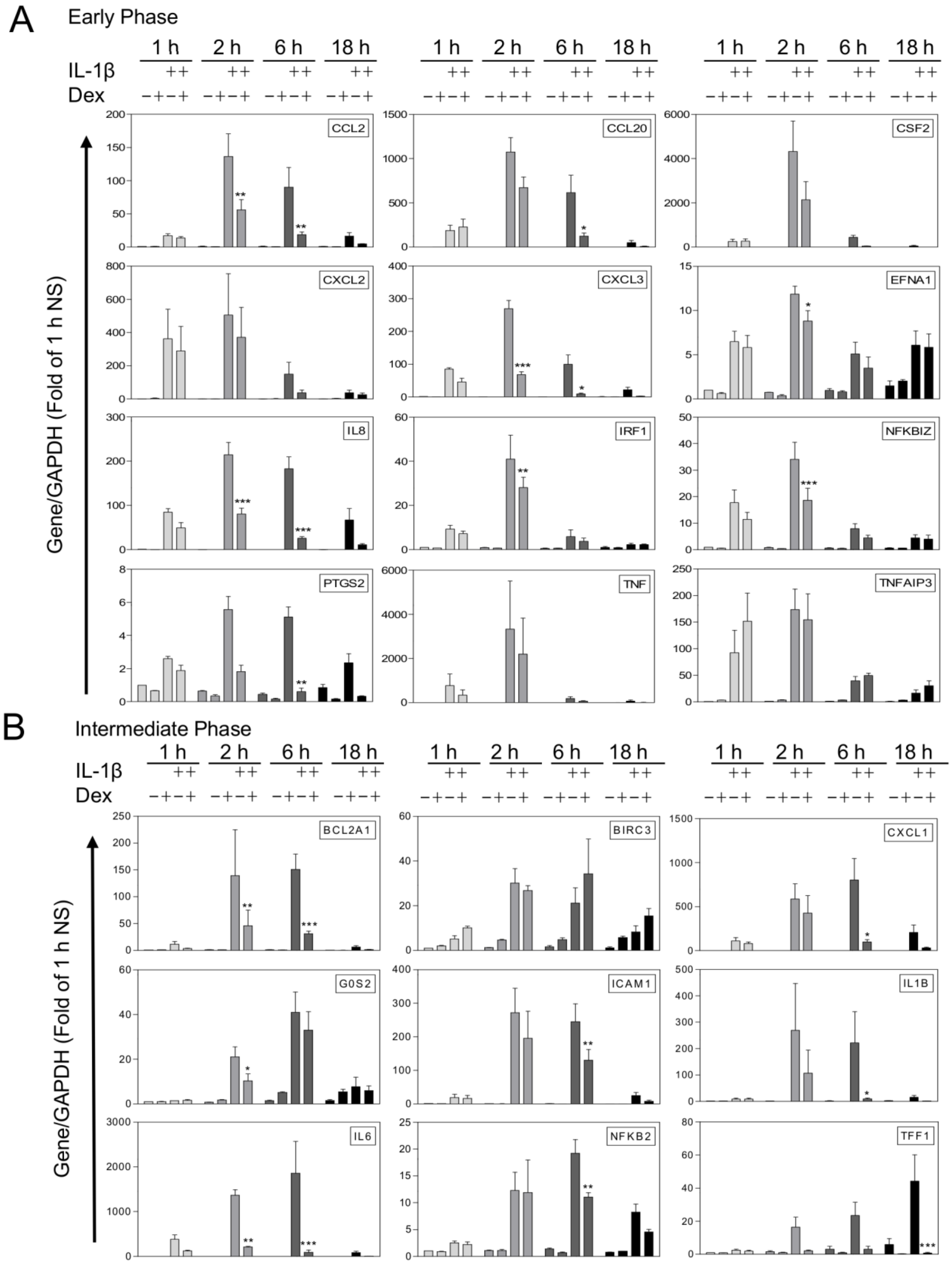

Supporting Figure S2 (continued)

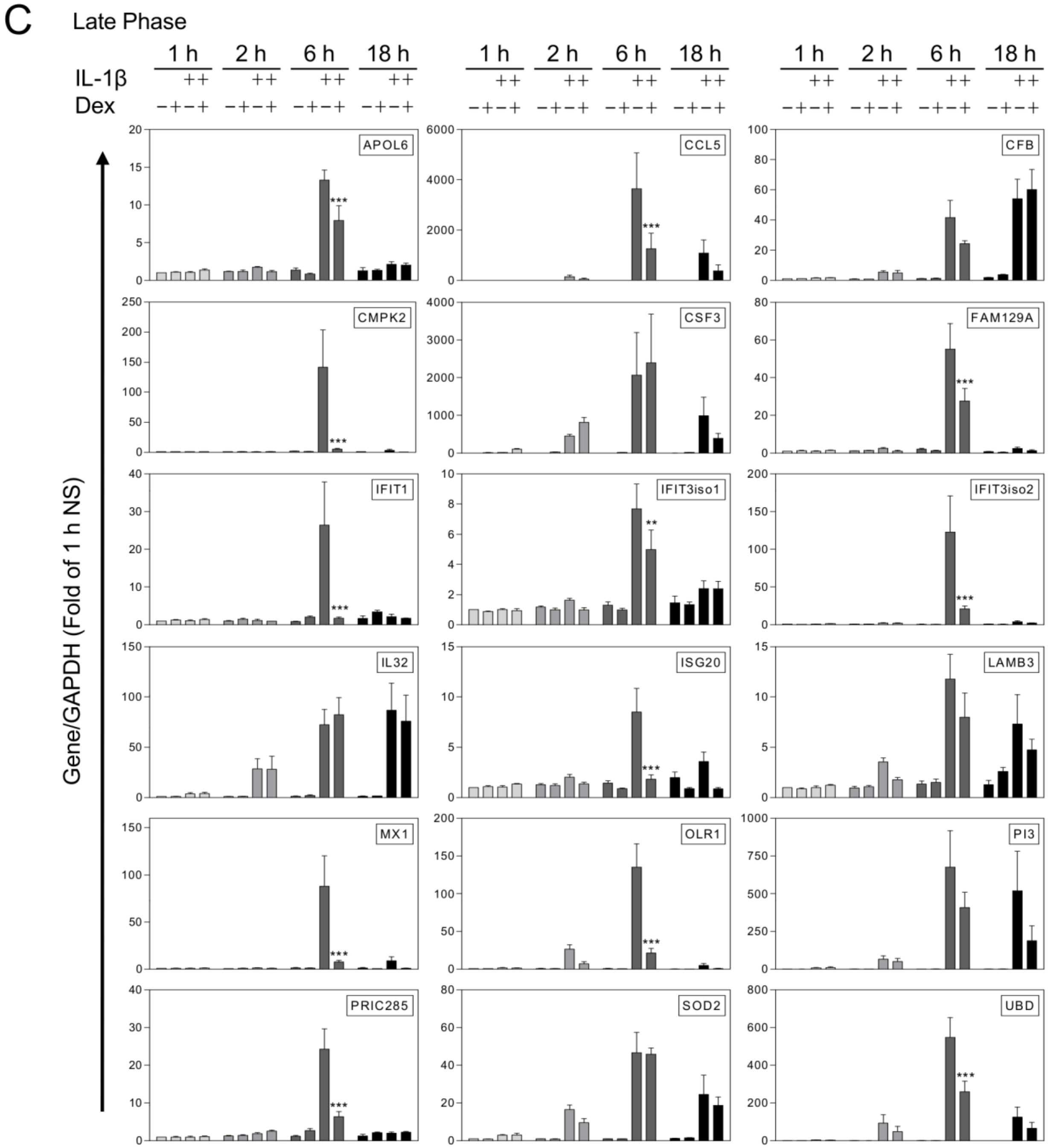

Supporting Figure S2. Time course analysis of inflammatory mRNA expression.
A549 cells were either not stimulated (NS) or treated with IL-1β (1 ng/ml), dexamethasone (Dex) (1 μM) or a combination of the two for 1, 2, 6 or 18 h. Cells were then harvested for RNA and real-time PCR was carried out for the indicated mRNAs and GAPDH. Data (n = 3) normalised to GAPDH and expressed as fold over NS at 1 h are plotted as mean ± SE. Genes are grouped based on expression patterns: (**A**) 'Early-phase' genes are those which have a peak of expression at 1 or 2 h; (**B**) 'Intermediate' genes have a peak of expression at 2, 6 or 18 h; (**C**) 'Late-phase' genes have a peak of expression at 6 h or later with less than 50 % of that peak expression observed at 1 or 2 h.

Significance relative to IL-1β treated samples at each time point was tested using ANOVA with a Bonferroni post-test and is indicated: *, $P < 0.05$; **, $P < 0.01$; ***, $P < 0.001$.
