# Supplementary material for: Glucocorticoid Repression of Inflammatory Gene Expression Shows Differential Responsiveness by Transactivation- and Transrepression-Dependent Mechanisms
Source: PLoS One. 2013 Jan 14;8(1):e53936. doi: 10.1371/journal.pone.0053936 (PMC3545719; doi:10.1371/journal.pone.0053936)
Supplement: Figure S3 — Effect of increasing concentrations of dexamethasone on IL-1β-induced inflammatory gene expression. A549 cells were either not stimulated (NS) or treated with IL-1β (1 ng/ml) and increasing concentrations of dexamethasone (as indicated) for 6 h prior to harvesting for RNA. Real-time PCR was carried out for GAPDH and the indicated genes. Data (n = 6) normalised to GAPDH and expressed as percentage of IL-1β are plotted as mean ± S.E. Significance relative to IL-1β-treated samples using ANOVA with a Dunnett’s post-test is indicated: *, P<0.05; **, P<0.01; ***, P<0.001. (PDF) [file pone.0053936.s003.pdf]

## Supporting Figure S3

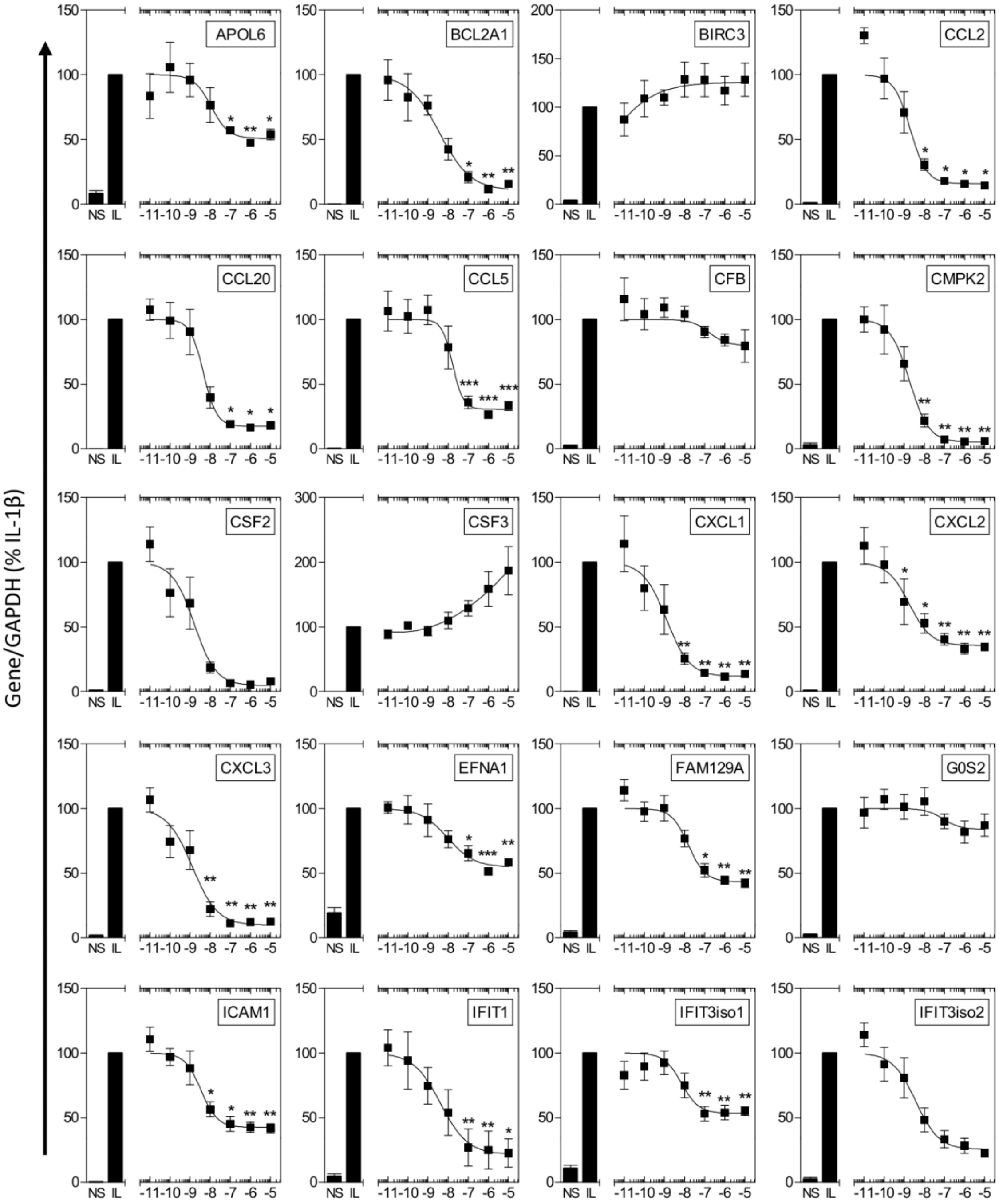

**Supporting Figure S3 (continued)**

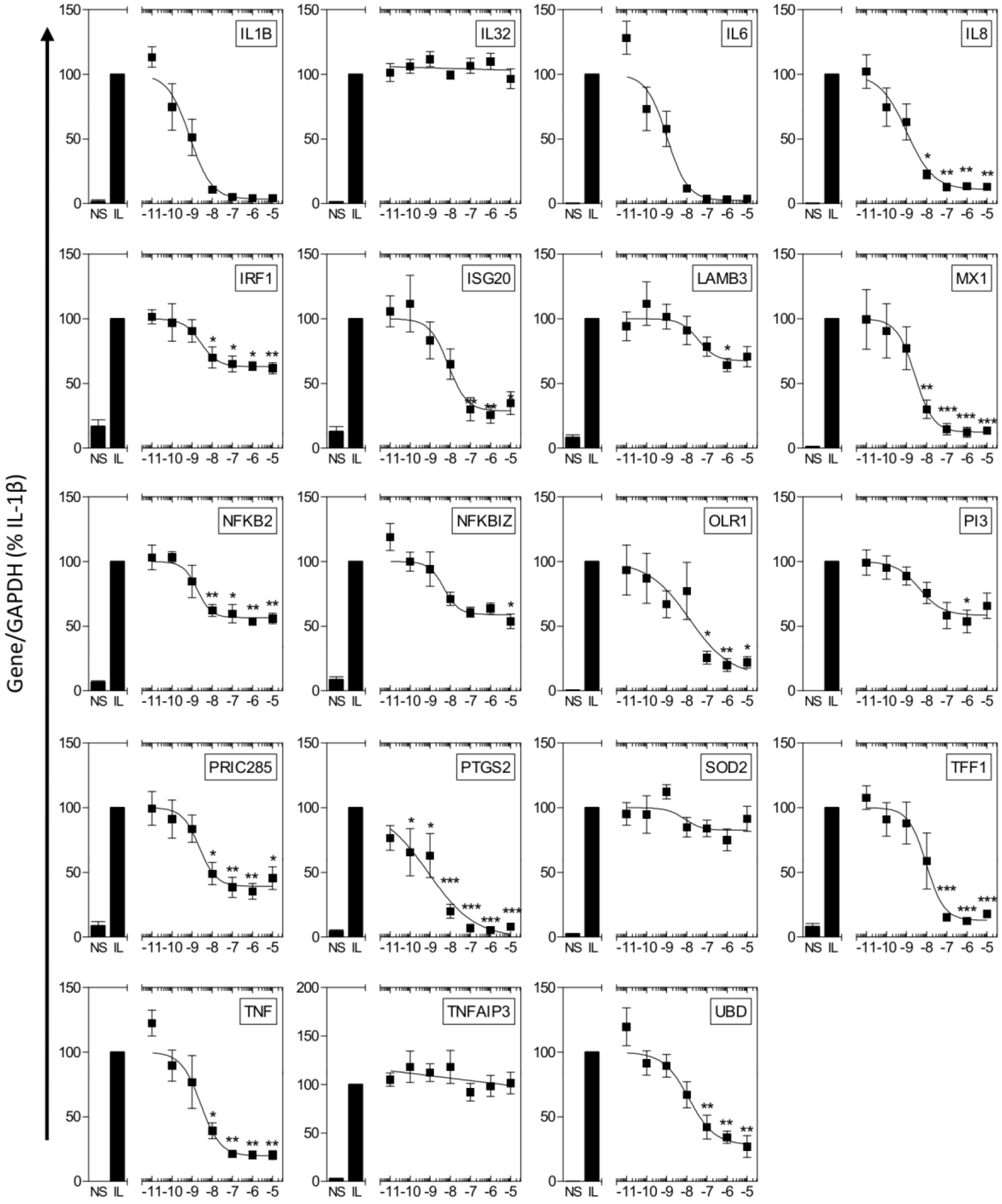


**Supplemental Figure S3. Effect of increasing concentrations of dexamethasone on IL-1β-induced inflammatory gene expression.**
A549 cells were either not stimulated (NS) or treated with IL-1β (1 ng/ml) and increasing concentrations of dexamethasone (as indicated) for 6 h prior to harvesting for RNA. Real-time PCR was carried out for GAPDH and the indicated genes. Data (n = 6) normalised to GAPDH and expressed as percentage of IL-1β are plotted as mean ± S.E. Significance relative to IL-1β-treated samples using ANOVA with a Dunnett's post-test is indicated: *, $P < 0.05$; **, $P < 0.01$; ***, $P < 0.001$.
