# Supplementary material for: Glucocorticoid Repression of Inflammatory Gene Expression Shows Differential Responsiveness by Transactivation- and Transrepression-Dependent Mechanisms
Source: PLoS One. 2013 Jan 14;8(1):e53936. doi: 10.1371/journal.pone.0053936 (PMC3545719; doi:10.1371/journal.pone.0053936)
Supplement: Figure S4 — Relationship between the effect of dexamethasone and Ad5-IκBαΔN. The effect of dexamethasone (1 µM) is plotted against the effect of Ad5-IκBαΔN (MOI 100), each following IL-1β treatment for 6 h and both expressed as percentage of IL-1β, for each mRNA. Data are derived from Figure 2A and 4C respectively. Linear regression was performed using GraphPad Prizm software. (PDF) [file pone.0053936.s004.pdf]

Supporting Figure S4

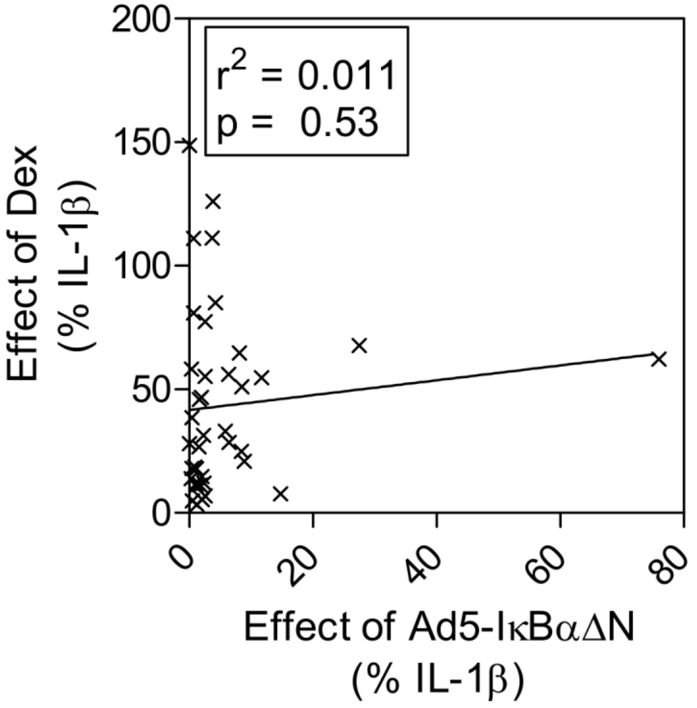

Supporting Figure S4. Relationship between the effect of dexamethasone and Ad5-IκBαΔN. The effect of dexamethasone (1 μM) is plotted against the effect of Ad5-IκBαΔN (MOI 100), each following IL-1β treatment for 6 h and both expressed as percentage of IL-1β, for each mRNA. Data are derived from Figure 2A and 4C respectively. Linear regression was performed using GraphPad Prizm software.
