# Supplementary material for: Glucocorticoid Repression of Inflammatory Gene Expression Shows Differential Responsiveness by Transactivation- and Transrepression-Dependent Mechanisms
Source: PLoS One. 2013 Jan 14;8(1):e53936. doi: 10.1371/journal.pone.0053936 (PMC3545719; doi:10.1371/journal.pone.0053936)
Supplement: Figure S5 — Effect of cycloheximide on IL-1β-induced inflammatory mRNA expression. A549 cells were treated with IL-1β (1 ng/ml) in the absence or presence of cycloheximide (CHX) (100 µg/ml) for 4 h. Cells were then harvested for real-time PCR analysis of the indicated genes and GAPDH. Data (n = 4) normalised to GAPDH and expressed as percentage of IL-1β treated samples are plotted as means ± SE. Significant repression relative to IL-1β treated samples was tested using a paired, one-way t-test and is indicated: *, P<0.05; **, P<0.01; ***, P<0.001. (PDF) [file pone.0053936.s005.pdf]

Supporting Figure S5

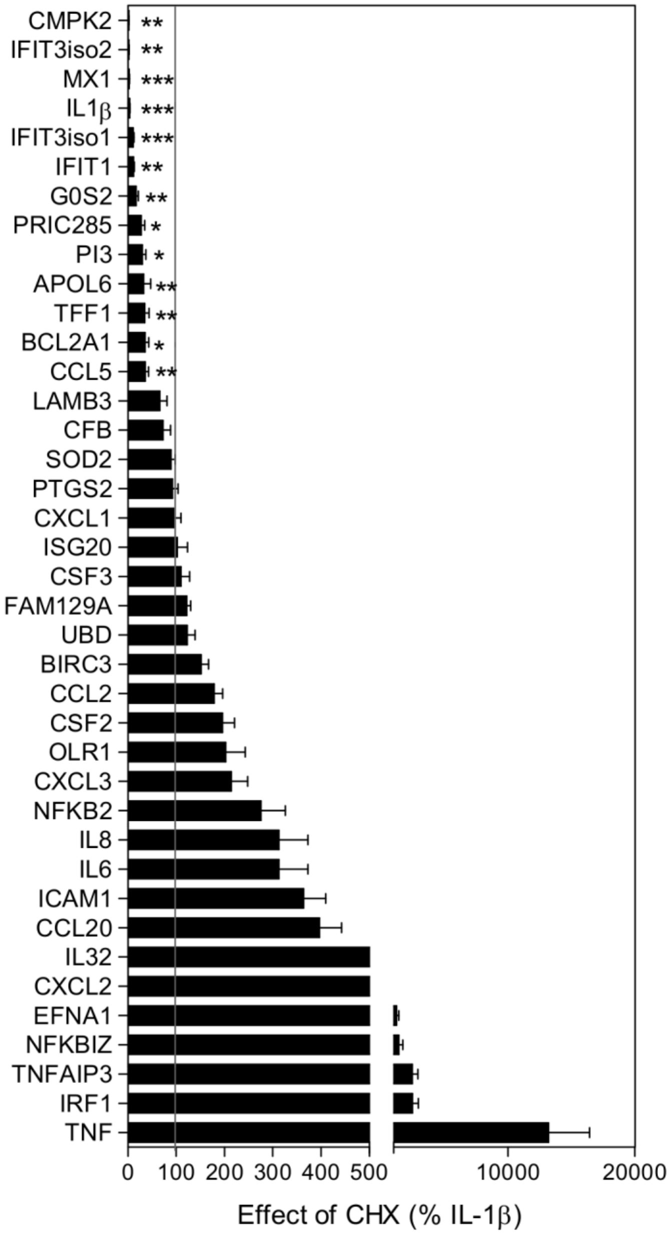

Supporting Figure S5. Effect of cycloheximide on IL-1β-induced inflammatory mRNA expression.

A549 cells were treated with IL-1β (1 ng/ml) in the absence or presence of cycloheximide (CHX) (100 µg/ml) for 4 h. Cells were then harvested for real-time PCR analysis of the indicated genes and GAPDH. Data (n = 4) normalised to GAPDH and expressed as percentage of IL-1β treated samples are plotted as means ± SE. Significant repression relative to IL-1β treated samples was tested using a paired, one-way t-test and is indicated: *, *P* < 0.05; **, *P* < 0.01; ***, *P* < 0.001.
