# Supplementary material for: Glucocorticoid Repression of Inflammatory Gene Expression Shows Differential Responsiveness by Transactivation- and Transrepression-Dependent Mechanisms
Source: PLoS One. 2013 Jan 14;8(1):e53936. doi: 10.1371/journal.pone.0053936 (PMC3545719; doi:10.1371/journal.pone.0053936)
Supplement: Table S1 — Primer sequences used in the study. Forward (F) and reverse (R) primer sequences (5′-3′) are shown in addition to the accession number for each gene. For genes with more than one RefSeq splice variant, primers were designed to amplify all variants, with the exception of IFIT3 for which two sets of primers were designed. (DOCX) [file pone.0053936.s006.docx]

**Supporting Table S1.** Primer sequences used in the study.

| **Target Gene** | **Accession Number** | **Primer Sequences** |
| --- | --- | --- |
| APOL6 | NM_030641.3 | F: CCCTGCCAGACCAGGGGACC  R: GGAGCGTCATCCTCATCCCTTTGC |
| BCL2A1 | NM_001114735.1; NM_004049.3 | F: CCCCGGATGTGGATACCTA  R: CTAGAAAAGTCATCCAGCCAGA |
| BIRC3 | NM_001165.3; NM_182962.1 | F: CCGTCAAGTTCAAGCCAGTTACCC  R: AGCCCATTTCCACGGCAGCA |
| CCL2 | NM_002982.3 | F: GCTCGCTCAGCCAGATGCAA  R: TCCTGAACCCACTTCTGCTTG |
| CCL5 | NM_002985.2 | F: TGCCTACATTGCCCGCCCAC  R: GGGTTGGCACACACTTGGCG |
| CCL20 | NM_001130046.1; NM_004591.2 | F: TGACATCAATGCTATCATCTTTCACA  R: TTTGCGCACACAGACAACTTTT |
| CFB | NM_001710.5 | F: ATGCCACATACCCCAAAATTTGGGT  R: GTTAGTCCCTGACTTCAACTTGTGGT |
| CMPK2 | NM_207315.2 | F: GCCGGGGCATGGAGAAGACC  R: TGGAGGGGCTGGCATCAACCA |
| CSF2 | NM_000758.2 | F: CCATGATGGCCAGCCACTACAAGC  R: ACTGGCTCCCAGCAGTCAAAGG |
| CSF3 | NM_000759.2; NM_172219.1; NM_172220.1 | F: AAGCTGTGCCACCCCGAGGA  R: GTGGGACCCAACTCGGGGGA |
| CXCL1 | NM_001511.2 | F: TCAATCCTGCATCCCCCA  R: CATAGAATCTTCAAAACTAATGAATAAAT |
| CXCL2 | NM_002089.3 | F: CGCATCGCCCATGGTTA  R: TAGAATCTTCTAAAACAAACAAATAAATA |
| CXCL3 | NM_002090.2 | F: TCATCGAAAAGATACTGAACAAGGG  R: GAAGTGTCAATGATACGCTGATAAGC |
| EFNA1 | NM_004428.2; NM_182685.1 | F: TCACAGTCCTCAGGCCCATGACA  R: GTGGGGCAGCACTGTGACCG |
| FAM129A | NM_052966.2 | F: GCTGGACGAGGGCAAGTGCG  R: AGGCGCCAATGGTGGCTTGG |
| GAPDH | NM_002046 | F: TTCACCACCATGGAGAAGGC  R: AGGAGGCATTGCTGATGATCT |
| G0S2 | NM_015714.3 | F: GCGCCGTGCCACTAAGGTCA  R: CACGCTGCCCAGCACGTACA |
| ICAM1 | NM_000201.2 | F: TGCCCTGATGGGCAGTCAACA  R: GCAGCGTAGGGTAAGGTTCTTG |
| IFIT1 | NM_001548.3 | F: AACCCTGCAGAACGGCTG  R: TGTAAAGTGACATCTCAATTGCTCC |
| IFIT3iso1 | NM_001549.4 | F: GCGTGCCCTACTCTCCCACC  R: AGCTGTGGAAGGATTTTCTCCAGGG |
| IFIT3iso2 | NM_001031683.2 | F: TCAGAACTGCAGGGAAACAGCCA  R: AGCTGTGGAAGGATTTTCTCCAGGG |
| IL1B | NM_000576.2 | F: TGGCAGAAGTACCTGAGCTCGC  R: GCCGCCATCCAGAGGGCAGA |
| IL6 | NM_000600.3 | F: CCTGAGAAAGGAGACATGTAACAAGA  R: GGAAGGTTCAGGTTGTTTTCTGC |
| IL8 | NM_000584.2 | F: GCAGCTCTGTGTGAAGGTGC  R: AAAGGTTTGGAGTATGTCTTTATGCA |
| IL32 | NM_001012631.1; NM_004221.4; NM_001012718.1; NM_001012636.1;  NM_001012635.1; NM_001012634.1; NM_001012632.1; NM_001012633.1 | F: GCAGCACCCAGAGCTCACTCC  R: AGGCTCCTCGGTTGCGGGAT |
| IRF1 | NM_002198.2 | F: CTCACTGCAGCCCCTGCGTC  R: TGGGCATGTTGGCTCTGCTGC |
| ISG20 | NM_002201.4 | F: TCCCTGCGGGTGCTGAGTGA  R: GCTCCATCGTTGCCCTCGCA |
| LAMB3 | NM_000228.2; NM_001127641.1; NM_001017402.1 | F: CAGCCAGGCTCCCCAACGTG  R: GGCTCGGCTCCTGGCTTCCT |
| MX1 | NM_001144925.1; NM_002462.3 | F: GGCAGCGGGATCGTGACCAG  R: CCTTCCCCGGCGATGGCATT |
| NFKB2 | NM_001077493.1; NM_002502.3; NM_001077494.1 | F: AACCCAAGGAGCCAGCCCCA  R: CAGCCATATCGAAATCGGAAG |
| NFKBIZ | NM_001005474.1; NM_031419.2 | F: GGCTTCTGGCCAAGCTGTGGAT  R: TCCCCGGGCGTTGGTGTTTG |
| OLR1 | NM_002543.3 | F: TGGTGCTGGGCATGCAATTATCCC  R: GCCGGGCTGAGATCTGTCCCT |
| PI3 | NM_002638.3 | F: AGCCTGGCTCCTGCCCCATT  R: GCAAGGACCGGCTCCCTCTCA |
| PRIC285 | NM_001037335.2; NM_033405.3 | F: ACGGTCATTCAGGGCCCACCA  R: GGCCTCAGCCTGCTCACTGT |
| PTGS2 | NM_000963.2 | F: GCTGGGCCATGGGGTGGACT  R: CCTGCCCCACAGCAAACCGT |
| SOD2 | NM_000636.2; NM_001024465.1; NM_001024466.1 | F: CGTGGCTGTGGTGGCTTCGG  R: CCTGCTGGTGCCGCACACT |
| TFF1 | NM_003225.2 | F: TCTGCGCCCTGGTCCTGGTG  R: GCACACTGGGAGGGCGTGAC |
| TNF | NM_000594.2 | F: GTGATCGGCCCCCAGAGGGAA  R: TGGAGCTGCCCCTCAGCTTGA |
| TNFAIP3 | NM_006290.2 | F: AGGCGCTGTTCAGCACGCTC  R: CGGGCCATGGGTGTGTCTGT |
| UBD | NM_006398.3 | F: GGTTTCTGGCCCCTTGTCTGCAG  R: ACGCTGTCATATGGGTTGGCATCA |

Forward (F) and reverse (R) primer sequences (5’-3’) are shown in addition to the accession number for each gene. For genes with more than one RefSeq splice variant, primers were designed to amplify all variants, with the exception of IFIT3 for which two sets of primers were designed.
