# Supplementary material for: Glucocorticoid Repression of Inflammatory Gene Expression Shows Differential Responsiveness by Transactivation- and Transrepression-Dependent Mechanisms
Source: PLoS One. 2013 Jan 14;8(1):e53936. doi: 10.1371/journal.pone.0053936 (PMC3545719; doi:10.1371/journal.pone.0053936)
Supplement: Table S3 — Effect of dexamethasone on inflammatory mRNA expression. The effect of dexamethasone (Dex) (1 µM) is shown on the induction of inflammatory mRNAs by IL-1β (1 ng/ml) at 6 h. The effect of dexamethasone is expressed as percentage of IL-1β treated. Data are derived from Figure 2A (Effect of Dex) and Figure S3 (EC50). There is no EC50 value available (n/a) for mRNAs that were not significantly repressed by dexamethasone. (DOCX) [file pone.0053936.s008.docx]

**Supporting Table S3.** Effect of dexamethasone on inflammatory mRNA expression.

| **Gene** | **Effect of Dex** | **Log EC_50_** |
| --- | --- | --- |
|  | **(% IL-1β)** |  |
| IL6 | 3.4 | -9.0 |
| IL1B | 4.9 | -9.1 |
| CMPK2 | 5.5 | -8.7 |
| CSF2 | 6.9 | -7.1 |
| PTGS2 | 7.8 | -9.1 |
| CXCL3 | 11 | -8.9 |
| MX1 | 11 | -8.6 |
| CXCL1 | 12 | -8.9 |
| TNF | 12 | -8.6 |
| IL8 | 14 | -8.9 |
| BCL2A1 | 15 | -8.4 |
| CCL2 | 18 | -8.7 |
| CCL20 | 18 | -8.3 |
| OLR1 | 19 | -7.9 |
| IFIT1 | 21 | -8.4 |
| ISG20 | 25 | -8.1 |
| IFIT3iso2 | 27 | -8.4 |
| CCL5 | 28 | -7.8 |
| TFF1 | 29 | -8.0 |
| CXCL2 | 31 | -8.8 |
| PRIC285 | 33 | -8.7 |
| UBD | 39 | -7.9 |
| ICAM1 | 46 | -8.5 |
| FAM129A | 47 | -7.8 |
| APOL6 | 51 | -7.9 |
| IFIT3iso1 | 55 | -8.1 |
| NFKB2 | 55 | -8.8 |
| EFNA1 | 56 | -8.0 |
| PI3 | 58 | -8.4 |
| NFKBIZ | 62 | -8.3 |
| LAMB3 | 65 | -7.4 |
| IRF1 | 68 | -8.6 |
| CFB | 77 | -6.8 |
| G0S2 | 81 | -8.8 |
| SOD2 | 85 | n/a |
| TNFAIP3 | 110 | n/a |
| IL32 | 110 | n/a |
| BIRC3 | 130 | n/a |
| CSF3 | 150 | n/a |

The effect of dexamethasone (Dex) (1 µM) is shown on the induction of inflammatory mRNAs by IL-1β (1 ng/ml) at 6 h. The effect of dexamethasone is expressed as percentage of IL-1β treated. Data are derived from Figure 2A (Effect of Dex) and Supplemental Figure S3 (EC_50_). There is no EC_50_ value available (n/a) for mRNAs that were not significantly repressed by dexamethasone.
