# Supplementary material for: Glucocorticoid Repression of Inflammatory Gene Expression Shows Differential Responsiveness by Transactivation- and Transrepression-Dependent Mechanisms
Source: PLoS One. 2013 Jan 14;8(1):e53936. doi: 10.1371/journal.pone.0053936 (PMC3545719; doi:10.1371/journal.pone.0053936)
Supplement: Table S4 — Effect of ORG34517 on IL-1β-induced gene expression and effect of lamin siRNA on dexamethasone-dependent repression of inflammatory mRNA expression. A. Effect of ORG34517 on inflammatory gene expression. A549 cells were either not stimulated or incubated with ORG34517 for 30 min prior to stimulation with IL-1β (1 ng/ml) for 6 h. RNA was extracted and real-time PCR analysis carried out for the indicated mRNAs. Data (n = 4–6) are expressed as percentage of IL-1β-treated samples. Statistical analysis was performed by paired t-test: *, P<0.05. B. Effect of lamin (control siRNA) on repression of inflammatory mRNA expression by dexamethasone. A549 cells were incubated with lamin siRNA for 24 h prior to stimulation with IL-1β (1 ng/ml) for 6 h in the absence or presence of dexamethasone (Dex) (1 or 0.1 µM). Data (n = 5) are expressed as percentage of IL-1β. Significance, relative to IL-1β+Dex was tested using ANOVA with a Dunnett’s post-test (see Figure 3D for other half of this analysis: IL-1β+Dex+lamin siRNA vs IL-1β+Dex+GR siRNA). ***, P<0.001. (DOCX) [file pone.0053936.s009.docx]

**Supporting Table S4.** Effect of ORG34517 on IL-1β-induced gene expression and effect of lamin siRNA on dexamethasone-dependent repression of inflammatory mRNA expression.

| **A** | **ORG data** | **B** | **GR siRNA data (10^-6^ M Dex)** | | **GR siRNA data (10^-7^ M Dex)** | |
| --- | --- | --- | --- | --- | --- | --- |
|  |  |  |  | **IL-1β + Dex** |  | **IL-1β + Dex** |
| **Gene** | **IL-1β + ORG** |  | **IL-1β + Dex** | **+ lamin siRNA** | **IL-1β + Dex** | **+ lamin siRNA** |
|  | **(% IL-1β)** |  | **(% IL-1β)** | **(% IL-1β)** | **(% IL-1β)** | **(% IL-1β)** |
| APOL6 | 110 ± 14 |  | 47 ± 4.3 | 47 ± 4.6 | 47 ± 5.4 | 47 ± 6.0 |
| BCL2A1 | 100 ± 8.4 |  | 25 ± 4.0 | 20 ± 2.8 | 26 ± 1.4 | 22 ± 2.3 |
| CCL2 | 77 ± 7.2 * |  | 16 ± 2.1 | 13 ± 1.7 | 16 ± 2.1 | 12 ± 1.4 |
| CCL20 | 110 ± 14 |  | 24 ± 6.0 | 24 ± 4.2 | 24 ± 6.2 | 24 ± 4.6 |
| CCL5 | 79 ± 8.9 |  | 37 ± 9.5 | 34 ± 7.6 | 27 ± 2.4 | 28 ± 4.9 |
| CFB | 84 ±7.0 |  | 85 ± 7.7 | 66 ± 8.0 | 85 ± 6.1 | 74 ± 3.8 |
| CMPK2 | 94 ± 22 |  | 10 ± 2.7 | 7.3 ± 1.3 | 11 ± 2.5 | 7.6 ± 1.6 |
| CSF2 | 100 ± 11 |  | 6.5 ± 1.6 | 6.0 ± 0.2 | 6.8 ± 1.3 | 6.0 ± 0.9 |
| CXCL1 | 78 ± 4.6 |  | 19 ± 6.7 | 21 ± 4.4 | 18 ± 4.5 | 17 ± 3.3 |
| CXCL2 | 100 ± 12 |  | 37 ± 3.1 | 36 ± 3.3 | 36 ± 0.5 | 33 ± 1.7 |
| CXCL3 | 88 ± 5.2 |  | 8.0 ± 2.0 | 5.2 ± 1.6 | 8.6 ± 3.1 | 5.4 ± 1.1 |
| EFNA1 | 90 ± 7.3 |  | 49 ± 5.0 | 48 ± 4.4 | 48 ± 3.4 | 43 ± 3.0 |
| FAM129A | 100 ± 9.4 |  | 39 ± 6.7 | 40 ± 5.4 | 46 ± 3.9 | 48 ± 5.6 |
| G0S2 | 98 ± 9.0 |  | 78 ± 12 | 59 ± 14 | 100 ± 16 | 56 ± 5.8 *** |
| ICAM1 | 110 ± 9.3 |  | 55 ± 6.5 | 59 ± 3.5 | 50 ± 2.3 | 56 ± 2.6 |
| IFIT1 | 78 ± 12 |  | 6.3 ± 2.1 | 4.4 ± 1.5 | 8.2 ± 3.6 | 4.9 ± 2.0 |
| IFIT3iso1 | 110 ± 11 |  | 36 ± 3.4 | 35 ± 3.7 | 45 ± 5.0 | 42 ± 5.0 |
| IFIT3iso2 | 120 ± 20 |  | 12 ± 3.7 | 9.4 ± 3.0 | 18 ± 8.6 | 13 ± 5.0 |
| IL1B | 83 ± 5.0 |  | 6.1 ± 2.3 | 4.9 ± 1.1 | 6.1 ± 2.1 | 4.7 ± 1.0 |
| IL6 | 97 ± 16 |  | 1.5 ± 0.4 | 1.7 ± 0.3 | 1.6 ± 0.5 | 1.7 ± 0.4 |
| IL8 | 82.4 ± 6.3 |  | 12 ± 2.0 | 12 ± 1.6 | 11 ± 1.0 | 11 ± 1.7 |
| IRF1 | 91 ± 9.8 |  | 44 ± 11 | 59 ± 11 | 45 ± 9.7 | 57 ± 13 |
| ISG20 | 92 ± 8.2 |  | 32 ± 9.9 | 34 ± 8.0 | 21 ± 3.5 | 22 ± 3.1 |
| LAMB3 | 90 ± 9.3 |  | 58 ± 8.9 | 70 ± 16 | 61 ± 5.9 | 70 ± 10 |
| MX1 | 97 ± 15 |  | 4.9 ± 1.3 | 4.8 ± 1.2 | 5.8 ± 1.6 | 5.4 ± 1.4 |
| NFKB2 | 110 ± 9.7 |  | 62 ± 8.6 | 67 ± 3.6 | 55 ± 3.6 | 58 ± 2.1 |
| NFKBIZ | 85 ± 4.1 |  | 55 ± 9.7 | 50 ± 7.4 | 49 ± 4.2 | 43 ± 3.5 |
| OLR1 | 93 ± 10 |  | 16 ± 2.3 | 11 ± 2.2 | 15 ± 2.1 | 9.7 ± 2.1 |
| PI3 | 87 ± 7.5 |  | 86 ± 2.5 | 78 ± 3.6 | 85 ± 4.8 | 71 ± 0.8 |
| PRIC285 | 120 ± 18 |  | 28 ± 7.8 | 29 ± 8.2 | 27 ± 6.8 | 31 ± 9.3 |
| PTGS2 | 92 ± 2.8 * |  | 7.2 ± 1.9 | 9.7 ± 3.0 | 6.4 ± 1.4 | 8.9 ± 3.6 |
| TFF1 | 67 ± 5.4 * |  | 7.0 ± 2.0 | 7.0 ± 1.9 | 5.9 ± 1.2 | 6.1 ± 1.3 |
| TNF | 97 ± 4.8 |  | 39 ± 12 | 40 ± 7.5 | 41 ± 5.2 | 45 ± 3.8 |
| UBD | 99 ± 6.1 |  | 32 ± 1.7 | 24 ± 2.9 | 37 ± 2.6 | 23 ± 2.2 |

**A.** Effect of ORG34517 on inflammatory gene expression. A549 cells were either not stimulated or incubated with ORG34517 for 30 min prior to stimulation with IL-1β (1 ng/ml) for 6 h. RNA was extracted and real-time PCR analysis carried out for the indicated mRNAs. Data (n = 4-6) are expressed as percentage of IL-1β-treated samples. Statistical analysis was performed by paired t-test: *, *P* < 0.05. **B.** Effect of lamin (control siRNA) on repression of inflammatory mRNA expression by dexamethasone. A549 cells were incubated with lamin siRNA for 24 h prior to stimulation with IL-1β (1 ng/ml) for 6 h in the absence or presence of dexamethasone (Dex) (1 or 0.1 µM). Data (n = 5) are expressed as percentage of IL-1β. Significance, relative to IL-1β + Dex was tested using ANOVA with a Dunnett’s post-test (see Figure 3D for other half of this analysis: IL-1β + Dex + lamin siRNA vs IL-1β + Dex + GR siRNA). ***, *P* < 0.001.
