# Supplementary material for: Glucocorticoid Repression of Inflammatory Gene Expression Shows Differential Responsiveness by Transactivation- and Transrepression-Dependent Mechanisms
Source: PLoS One. 2013 Jan 14;8(1):e53936. doi: 10.1371/journal.pone.0053936 (PMC3545719; doi:10.1371/journal.pone.0053936)
Supplement: Materials and Methods S1 — Additional materials and methods. (DOCX) [file pone.0053936.s010.docx]

**Supporting Information**

**Glucocorticoid repression of inflammatory gene expression shows differential responsiveness by transactivation- and transrepression-dependent mechanisms**

**Elizabeth M. King^1^, Joanna E. Chivers^1^, Christopher F. Rider^1^, Anne Minnich^2^, Mark A. Giembycz^1^, Robert Newton^1^**

**1** Airways Inflammation Research Group, Snyder Institute for Chronic Diseases, Faculty of Medicine, University of Calgary, Calgary, Alberta, Canada, **2** Clinical Biomarkers Immunology, Bristol-Myers Squibb, Princeton, New Jersey, USA

**Corresponding author:** Dr. Robert Newton

Department of Cell Biology and Anatomy

Faculty of Medicine

University of Calgary

3330 Hospital Drive NW

Calgary, AB, T2N 4N1, Canada

Tel: 001 403 210 3938

Fax: 001 403 270 8928

e-mail: [rnewton@ucalgary.ca](mailto:rnewton@ucalgary.ca)

**Additional Materials and Methods**

Microarray analysis

A549 cells were either not stimulated or treated with IL-1β (1 ng/ml), dexamethasone (1 µM) or a combination of the two for 6 or 18 h (n = 3). Total RNA was prepared as described and assayed for quality using RNA LabChips (Agilent Technologies, Palo Alto, CA). Initially, experiments were validated by performing northern blotting for COX-2 and IL-8 to confirm up-regulation by IL-1β and repression by dexamethasone (data not shown). Subsequently, RNA (5 µg) was reverse transcribed to cDNA prior to being transcribed in vitro to generate biotin-labelled cRNA before fragmentation and hybridisation with the GeneChip human genome U95Av2 and B arrays as specified by the manufacturer (Affymetrix Inc., Santa Clara, CA). The array was subsequently washed and stained with a strepdavidin-phycoerythrin-conjugated anti-biotin to visualise hybridised cRNA and then the GeneChip was scanned to quantify gene expression. After global normalisation, analysis was performed using the P-FOLD algorithm for Bayesian estimation of fold changes [1]. Microarray analysis was performed by Aventis at the Cambridge Genomics Center, Bridgewater, NJ, USA.

**References**

1 Theilhaber, J., Bushnell, S., Jackson, A., and Fuchs, R. (2001) Bayesian estimation of fold-changes in the analysis of gene expression: the PFOLD algorithm J. Comput. Biol. **8**, 585-614
